# Achieving international biodiversity targets: Learning from local norms, values and actions regarding migratory waterfowl management in Kazakhstan

**DOI:** 10.1111/1365-2664.14198

**Published:** 2022-05-30

**Authors:** Isabel L. Jones, Alexey Timoshenko, Ivan Zuban, Konstantin Zhadan, Jeremy J. Cusack, A. Bradley Duthie, Isla D. Hodgson, Jeroen Minderman, Rocío A. Pozo, Robin C. Whytock, Nils Bunnefeld

**Affiliations:** ^1^ Biological and Environmental Sciences University of Stirling Stirling UK; ^2^ Kazakhstan Association for the Conservation of Biodiversity (ACBK) Nur‐Sultan Kazakhstan; ^3^ M. Kozybayev North Kazakhstan State University Petropavlovsk Kazakhstan; ^4^ Centre for Ecosystem Modelling and Monitoring Universidad Mayor Santiago Chile; ^5^ Faculty of Agronomy Pontifical Catholic University of Valparaíso Quillota Chile

**Keywords:** biodiversity targets, conservation conflict, ecological modelling, global to local, hunting, migratory species, policy‐making, socio‐ecological surveys

## Abstract

1. Migratory species are protected under international legislation; their seasonal movements across international borders may therefore present opportunities for understanding how global conservation policies translate to local‐level actions across different socio‐ecological contexts. Moreover, local‐level management of migratory species can reveal how culture and governance affects progress towards achieving global targets. Here, we investigate potential misalignment in the two‐way relationship between global‐level conservation policies (i.e. hunting bans and quotas) and local‐level norms, values and actions (i.e. legal and illegal hunting) in the context of waterfowl hunting in northern Kazakhstan as a case‐study.
2. Northern Kazakhstan is globally important for waterfowl and a key staging area for arctic‐breeding species. Hunting is managed through licences, quotas and seasonal bans under UN‐AEWA intergovernmental agreements. To better understand the local socio‐ecological context of waterfowl hunting, we take a mixed‐methods approach using socio‐ecological surveys, informal discussions and population modelling of a focal migratory goose species to: (a) investigate motivations for hunting in relation to socio‐economic factors; (b) assess knowledge of species' protection status; and (c) predict the population size of Lesser White‐fronted Geese (LWfG; *Anser erythropus*; IUCN Vulnerable) under different scenarios of survival rates and hunting offtake, to understand how goose population demographics interact with the local socio‐ecological context.
3. Model results showed no evidence that waterfowl hunting is motivated by financial gain; social and cultural importance were stronger factors. The majority of hunters are knowledgeable about species' protection status; however, 11% did not know LWfG are protected, highlighting a key area for increased stakeholder engagement.
4. Simulations of LWfG population growth over a 20‐year period showed LWfG are highly vulnerable to hunting pressure even when survival rates are high. This potential impact of hunting highlights the need for effective regulation along the entire flyway; our survey results show that hunters were generally compliant with newly introduced hunting regulations, showing that effective regulation is possible on a local level.
5. *Synthesis and applications*. Here, we investigate how global conservation policy and local norms interact to affect the management of a threatened migratory species, which is particularly important for the protection and sustainable management of wildlife that crosses international borders where local contexts may differ. Our study highlights that to be effective and sustainable in the long‐term, global conservation policies must fully integrate local socio‐economic, cultural, governance and environmental contexts, to ensure interventions are equitable across entire species' ranges. This approach is relevant and adaptable for different contexts involving the conservation of wide‐ranging and migratory species, including the 255 migratory waterfowl covered by UN‐AEWA (United Nations Agreement on the Conservation of African‐Eurasian Migratory Waterbirds).

## Graphical abstract

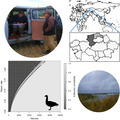

## Введение

1. Мигрирующие виды находятся под охраной международного законодательства; их сезонные перемещения через международные границы могут предоставить возможности для понимания того, как глобальная природоохранная политика преобразуется в действия на уровне регионов в различных социально‐экологических аспектах. Более того, региональный менеджмент мигрирующих видов может показать, как культура и административное руководство влияют на прогресс в достижении глобальных целей. Здесь в качестве кейса мы исследуем потенциальную несогласованность во взаимосвязи глобальной природоохранной политики (к примеру, запретами на охоту и квоты) и региональными нормами, ценностями и действиями (к примеру, законной охотой и браконьерством) в контексте охоты на водоплавающую дичь в Северном Казахстане.
2. Северный Казахстан имеет глобальное значение для водоплавающих птиц и является ключевым перевалочным пунктом для гнездящихся видов в Арктике. Регуляция охоты осуществляется через лицензии, квоты и сезонные запреты в соответствии с межправительственными соглашениями ООН‐AEWA. Чтобы лучше понять местный социально‐экологический аспект охоты на водоплавающих птиц, мы применили подход со смешанными методами, используя социально‐экологические опросы, неофициальные обсуждения и моделирование популяции одного из основных мигрирующих видов гусей, чтобы: (a) выявить мотивы к охоте в зависимости от социально‐экономических факторов; (b) оценить знания о статусе охраны видов; и (c) спрогнозировать численность популяции гусей‐пискулек (*Anser erythropus*; Уязвимый вид МСОП) при различных сценариях выживаемости и добычи на охоте, чтобы понять, как демография популяции гусей перекликается с местной социально‐экологической обстановкой.
3. Результаты моделирования не показали никаких доказательств того, что охота на водоплавающих птиц мотивирована финансовой выгодой; социальная и культурная значимость были более сильными факторами. Большинство охотников осведомлены о статусе охраны видов; несмотря на это, 11% не знали, что гусь‐пискулька находятся под защитой, что подчеркивает ключевую область, требующую повышения вовлеченности стейкхолдеров.
4. Моделирование роста популяции гуся‐пискульки за 20‐летний период показало, что этот вид очень уязвим к прессингу охоты, даже когда показатели выживаемости высоки. Это потенциальное воздействие охоты подчеркивает необходимость эффективного регулирования вдоль всего пролетного пути; результаты нашего опроса показывают, что охотники в целом соблюдают недавно введенные правила охоты, демонстрируя то, что эффективное регулирование на региональном уровне является возможным.
5. *Синтез и приложения*. Здесь мы исследуем, как взаимодействуют глобальная природоохранная политика и региональные нормы, для воздействия на управление мигрирующими видами, находящимися под угрозой исчезновения, что особенно важно для защиты и устойчивого менеджмента дикой природы, пересекающей международные границы, где региональные условия могут отличаться. Наше исследование подчеркивает, что для того, чтобы быть эффективной и устойчивой в долгосрочной перспективе, глобальная природоохранная политика должна полностью учитывать региональные социально‐экономические, культурные, управленческие и экологические аспекты, чтобы обеспечить справедливый характер вмешательств во всех ареалах обитания видов. Этот подход актуален и может быть адаптирован к различным аспектам, связанным с сохранением широко распространенных и мигрирующих видов, включая 255 мигрирующих водоплавающих птиц, охваченных программой ООН‐AEWA.

## 1 INTRODUCTION

### 1.1 Global to local migratory waterfowl conservation

The conservation of migratory species is an international priority. Intergovernmental agreements including the United Nations Convention on the Conservation of Migratory Species of Wild Animals (CMS, 1979) are in place to coordinate conservation efforts across species' ranges. Migratory birds are threatened by over‐harvesting, and environmental and land‐use change (Runge et al., 2015). Targeted intergovernmental agreements, such as the United Nations Agreement on the Conservation of African‐Eurasian Migratory Waterfowl (UN‐AEWA, 2018) bring together countries and conservation organisations to coordinate conservation and management across flyways (Madsen et al., 2017). Through such agreements, global‐level conservation targets are delivered via local‐level actions, including species monitoring, habitat conservation, education and hunting management to maintain conservation status (Cusack et al., 2019; UN‐AEWA, 2018).

Top‐down conservation decisions and interventions may be misaligned with local norms, values and actions (Moilanen & Arponen, 2011). For instance, killing a particular species may be illegal, but if that species damages local peoples' livelihoods, or if harvesting is crucial for subsistence, then illegal killing may occur (Whytock et al., 2018). Governments and statutory bodies are under increasing societal and political pressure to resolve environmental problems with limited resources (Young et al., 2016). Therefore ‘solutions’ often focus on technical, monetary or legislative mechanisms and lack in‐depth analyses of local social and cultural contexts (Hodgson et al., 2020). Scenarios of global‐level policies being applied to different local socio‐economic, environmental and governance contexts can lead to conservation conflicts, in which stakeholder groups clash over objectives (e.g. hunting rights) and one group asserts its interests over those of another (e.g. hunting bans; Cusack et al., 2020; Redpath et al., 2013).

To manage such conflicts in an effective way, it is critical to understand the multidimensional context in which they are situated (Young et al., 2020). Recent studies have engaged stakeholders to understand perceptions and motivations for particular actions to identify mutually beneficial conservation interventions for stakeholders and biodiversity (e.g. Rakotonarivo et al., 2020, 2021). The challenge of managing conservation conflicts is compounded when they cross international borders and involve diverse stakeholders and local contexts, as is the case for many wide‐ranging or migratory species. Thus, to achieve effective conservation outcomes, better integration of global and local contexts is needed.

### 1.2 Global conservation policies in the local context of northern Kazakhstan

We use waterfowl hunting in northern Kazakhstan as a case study to understand the conflicts and synergies between global conservation policy and local‐level actions. Northern Kazakhstan is a key staging ground for arctic‐breeding migrant waterfowl, including the Lesser White‐fronted Goose (LWfG; *Anser erythropus*; IUCN Vulnerable; estimated population size <40,000; Cuthbert et al., 2018). Northern Kazakhstan is the focus of global‐level policies and intergovernmental conservation efforts under UN‐AEWA: hunting waterfowl, including certain migratory species, is restricted with offtake managed through hunting quotas.

Aligning with UN‐AEWA policies, Kazakhstan has red listed the globally declining LWfG—the focal species of this study—making hunting LWfG illegal. However, illegal hunting in Kazakhstan and across the flyway may be a key threat to LWfG, jeopardising the efficacy of conservation interventions (Jones et al., 2008). Hunting is culturally and socially important, and there are between 10,000 (Yerokhov, 2013) and 15,000 (unpublished data) licensed hunters across northern Kazakhstan. Thus, top‐down implementation of hunting bans or restrictions may conflict with local norms and values, potentially leading to social disengagement with conservation goals, unknown levels of illegal offtake, and consequent uncertain LWfG population and conservation outcomes.

Here, we aim to illustrate how effective global conservation policies rely on a close understanding of the complex two‐way relationship between local socio‐ecological contexts and global policies. To this end, we use waterfowl hunting in northern Kazakhstan, and specifically LWfG, as a case study. Using a mixed‐methods approach including socio‐ecological surveys and informal discussions with local people, alongside population modelling, we aim to: (a) investigate motivations for waterfowl hunting in relation to socio‐economic factors; (b) assess knowledge of waterfowl species protection status and (c) predict population sizes of LWfG under different scenarios of survival rates and hunting offtake. This multidisciplinary approach allows greater understanding of how the local socio‐ecological context and uncertainty in population parameters (including unknown levels of illegal hunting) may affect LWfG conservation outcomes, and illuminates the complex two‐way relationship between local contexts and global policies.

## 2 MATERIALS AND METHODS

### 2.1 Study area

**FIGURE 1:**
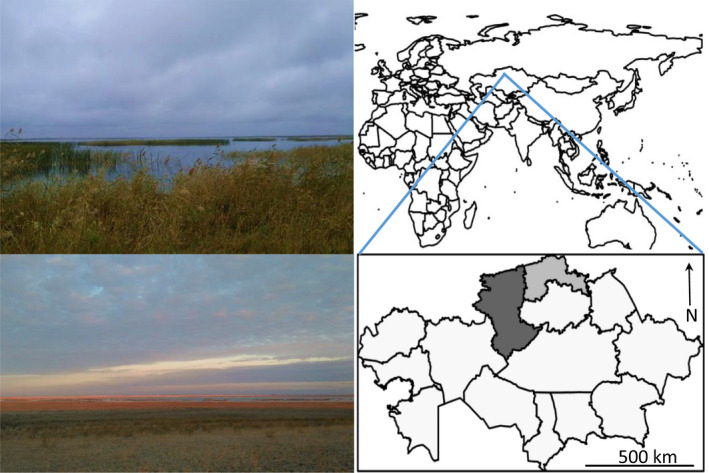
Geography and landscape of northern Kazakhstan, comprising a mosaic of wetlands, steppe and forest‐steppe. Inset map depicts the 14 regions of Kazakhstan. The two regions where socio‐ecological surveys were conducted are highlighted: the Kostanay region (dark grey) and North Kazakhstan (light grey). Map created using GADM (Global Administrative Areas, 2017) and r package ‘sp’ (Bivand et al., 2017)

The northern Kazakhstan landscape comprises a mosaic of agriculture, forest‐steppe and steppe with numerous lakes and wetlands (Figure 1), which are among the most extensive and important in Central Asia for migratory waterfowl (Kamp et al., 2015; Yerokhov, 2006). Millions of arctic‐breeding waterfowl use the region as a key staging area over a 3–5 week period in autumn and in spring (Cuthbert et al., 2018; Zuban et al., 2020). Our study focuses on two regions, the Kostanay region and North Kazakhstan, which contain key waterfowl staging areas (Figure 1). Rural human population density is low, with scattered small villages and isolated homesteads. The latest census figures (ASPR, 2021) indicate total populations of 863,290 (Kostanay Region; 196,000 km^2^) and 542,500 (North Kazakhstan; 98,000 km^2^). Census figures include the regional capital cities, Kostanay and Petropavl.

#### 2.1.1 Waterfowl hunting in northern Kazakhstan

Hunting is socially and culturally important: there are approximately 10,000 licensed hunters across the two study regions, many belonging to rural hunting clubs that also host international visiting hunters (Jones et al., 2017; Yerokhov, 2013). Waterfowl hunting is permitted between September and November, coinciding with the autumn migration; a spring hunting ban between March and May was implemented in 2017 to protect birds on their return migration. The main legal quarry species of geese are Greylag Geese *Anser anser* and Greater White‐fronted Geese *Anser albifrons*, of which the latter looks very similar to LWfG (see Figure S1 for main quarry species detailed on hunting licences). LWfG fly in mixed flocks with Greater White‐fronted Geese and Greylag Geese (Cuthbert & Aarvak, 2016). There is therefore the possibility of misidentification of LWfG in mixed flocks and accidental illegal offtake by licensed hunters, as well as illegal offtake by unlicensed hunters (Yerokhov, 2013).

Regional hunting quotas can vary among years and are set by local authorities based on their annual species counts. Licences cost between US$25–50 (monthly salaries in rural areas range between US$100–170). Licences vary in cost depending on both the species listed and the hunting quota. Illegal hunting (e.g. over quota or targeting species not included on a licence) is punishable by fines or imprisonment if caught hunting a red listed species. Regional inspectors monitor compliance, but enforcement efficacy can be limited by the size of area covered by single inspectors (~3,000 ha), and limited legal powers to search hunting bags. Hunting inspectors can be hunters themselves, and thus enforcement of hunting regulations can be complex and challenging (Jones et al., 2017).

#### 2.1.2 The Lesser White‐fronted Goose (LWfG)

LWfG breed discontinuously from Scandinavia to eastern Siberia. LWfG migration routes, from northern breeding grounds to southern wintering grounds (northeast Greece, Iraq, southwest of the Caspian Sea and southeast China) via key staging areas in Central Asia and eastern Europe, are only partially known (Jones et al., 2008). The global LWfG population has declined from an estimated 250,000–300,000 in the 19th Century (Mooij, 2010) to recent estimates of <40,000 individuals (Cuthbert et al., 2018). LWfG are IUCN ‘Vulnerable’ and covered by a UN‐AEWA Single Species Action Plan: loss and fragmentation of breeding grounds, and hunting at staging areas, are thought to be key threats to the LWfG population (Jones et al., 2008).

Staging areas in northern Kazakhstan are particularly important for LWfG because the entire Western main subpopulation (recently estimated at 32,000 [25,400–38,700; 95% C.I.; Cuthbert & Aarvak, 2016]) as well as individuals from the critically endangered Fennoscandian subpopulation, migrate through the area in autumn with newly‐fledged offspring (Cuthbert et al., 2018). Moreover, the Western main subpopulation migrates back through in spring (Jones et al., 2008; Zuban et al., 2020).

### 2.2 Socio‐ecological surveys

Hunting, and especially illegal hunting, can be highly sensitive topics among hunters and the public due to social and cultural norms. In order to investigate motivations for waterfowl hunting, we used a mix of ‘specialised questioning techniques’ (Nuno & St. John, 2015) specifically the unmatched count technique (UCT; Droitcour et al., 1991); direct questioning using a questionnaire; followed by informal discussions to gain broader contextual information (Young et al., 2018). We developed all questioning techniques with our local partner, the Kazakhstan Association for the Conservation of Biodiversity (ACBK), to ensure relevance and appropriateness to the local socio‐ecological context.

Surveys were undertaken during the autumn waterfowl hunting season in September and October 2017 throughout the Kostaney region and North Kazakhstan (Figure 1). Respondents were recruited opportunistically in 46 spatially independent sites, comprising villages and isolated homesteads, lakes and agricultural land, and at hunting clubs via a pre‐arranged member gatherings (hereafter referred to as ‘site’). ‘Site’ areas cannot be defined precisely but were in the order of 1 km^2^, separated by a minimum of 5 km (mean distance between ‘sites’ was approximately 45 km). Respondents did not need to be hunters to participate in the survey because we were interested to know how common species protection knowledge is. Discussions were solicited following questionnaire surveys: bilingual co‐authors facilitated communication between the lead author and respondents.

#### 2.2.1 Motivations for waterfowl hunting: The Unmatched Count Technique (UCT)

The UCT is a useful method to ask questions about sensitive topics, including illegal hunting (Hinsley et al., 2019; Nuno & St. John, 2015). Participants are shown a list of images of different activities they may undertake and are asked how many they have undertaken: the number stated is the response variable in statistical analysis. ‘Control’ and ‘Treatment’ lists both contain the same four non‐sensitive activities; the treatment list includes an additional image depicting the sensitive activity. In this study, non‐sensitive activities were ‘farming or herding’, ‘driving a taxi’, ‘construction’ and ‘milking cows’. These activities were selected with ACBK to ensure each activity had the potential to be undertaken by participants. Both common and less common activities to men and women were included (Tsuchiya et al., 2007; sample UCT picture cards are included in Appendix S1).

During survey development with ACBK, we decided that in this particular context, mentioning LWfG in relation to hunting was too sensitive (because it is a red listed species and its hunting carries severe penalties) with a high risk of participants refusing to engage with the questionnaire. Thus, to ensure engagement was maximised, the UCT focused on hunting geese in general, without detailing a specific species. By doing so, we could use ‘hunting geese’ as the sensitive activity because hunting is illegal without a licence, and because a spring hunting ban had been newly implemented.

Participants were randomly assigned to ‘Control’ or ‘Treatment’ groups and were asked a training question (how many activities have you undertaken in the past month?) to ensure the UCT was understood (data not analysed). Participants were then asked: (a) How many of these activities have you done in the past year?; (b) How many activities have you done in autumn/winter (September–February); (c) How many of these activities have you done in spring/summer (March–August)?; (d) How many of these activities have you done for cash? (Appendix S1).

#### 2.2.2 Demographics, hunting licence ownership and species protection knowledge

Information on participant demographics, hunting licence ownership and knowledge of waterfowl species protection status was obtained through a questionnaire (Appendix S1). We assessed whether survey respondents knew if a selection of waterfowl species were protected or not (species were selected with ACBK to include LWfG and four other protected and non‐protected species): correct answers were awarded one point and points were summed to give an overall knowledge score for each respondent.

### 2.3 Ethics statement

This study was reviewed by the General University Ethics Panel (University of Stirling; GUEP262). Key ethical considerations included complete anonymisation of data, including survey locations (St. John et al., 2016). Field research was conducted in partnership with ACBK (research permit number unavailable).

### 2.4 Socio‐ecological survey data analysis

We excluded data from women from statistical analyses because in our study area, hunting was exclusively conducted by men (final sample size *n* = 166). Predictor variables (Table S1) were inspected for co‐linearity and for any pair of variables with *r* > 0.6, the variable of most relevance to the question was retained. Hunting licence types were catagorised into ‘goose and duck’ or ‘single species group’ licences.

Data were analysed using (generalised) linear mixed‐effects models ([G]LMMs) using the ‘lme4’ package (Bates et al., 2015) in ‘r’ (R Core Team, 2020) and 95% confidence intervals for parameter estimates were generated by bootstrapping (1,000 samples using ‘bootMer’ [‘lme4’ package]).

#### 2.4.1 Motivations for waterfowl hunting (UCT data)

Using ‘ict.test’ (r package ‘list’; Blair & Imai, 2010), we found no evidence of a design effect (*p* values between 0.31–1 for each list item; Whytock et al., 2018). The number of activities undertaken was modelled using LMMs with demographic variables, degree of species protection knowledge and licence type as predictors (Table S1). Interactions between predictor variables and ‘group’ (control or treatment) were used to examine the effect of all predictor variables, including the sensitive activity in the treatment group. A ‘long list’ of potential models was defined as all possible combinations of predictors, but only including a given main effect when it was also included as an interaction with treatment (because for the purposes of this study, we were only interested in the treatment interaction effects with each predictor), and always including treatment as a predictor. All full candidate sets also included an ‘intercept only’ model, and all had the same random effect structure (single random intercept for ‘site’). Models were compared using AICc scores, and those within ΔAICc < 4 of the top model were taken as the top model set. A ‘final’ model was then defined as one including all fixed effect predictors/interactions in this top set, and used for interpretation. Additionally, we present model‐averaged parameter estimates based on the ΔAICc < 4 set, using the natural averaging method.

#### 2.4.2 Species protection knowledge

Species protection scores were analysed using a GLMM (Poisson error structure) with demographic variables and licence type (Table S1). Knowledge of LWfG protection was analysed using a GLMM (Binomial error structure). Models were compared using the same procedure as for the UCT data analysis above (excluding treatment and treatment interaction terms as these are not relevant for these data); we present both a final model including all predictors in the top set, as well as model‐averaged estimates.

### 2.5 LWfG population modelling

Using demographic parameters sourced from empirical literature, we constructed a simple population model to numerically simulate the effect of varying levels of illegal hunting on LWfG population dynamics and extinction probability over a 20‐year period. In a single year of our model, the LWfG population size observed in the study area at time *t* + 1, *N*_*t*+1_, is the result of three processes: (a) the survival and return of individuals present in the study area at year *t* (hereafter referred to as the ‘return rate’); (b) the recruitment into the population of a fixed number of juveniles accompanying returning individuals; and (c) a fixed carrying capacity effect, which increases the probability of individual mortality linearly whenever population size exceeds carrying capacity. Illegal harvesting takes place once all individuals have returned to the study area. This assumes that mortality along the rest of the flyway, reflected in the return rate, and mortality due to illegal hunting are additive.

Adult return rate was modelled using *N*_*t*_ independent Bernoulli trials, such that the total number of adults returning to the study area at *t* + 1 (*N*_*a*,*t*+1_) was modelled by sampling from a binomial distribution,

$$N_{a,t+1}\sim \text{Binomial}\left(N_{t},\phi \right)$$

in which ϕ is the mean individual return rate. Note that this assumes mortality along the flyway is independent across all individuals (Schmutz & Ely, 1999). Of the surviving individuals who return to the study area, we assume 50% are female, and that of these, only a proportion *p* is accompanied by offspring recruited during the breeding season (i.e. *p* is the proportion of returning females who successfully breed). The number of juveniles recruited into the population at *t* + 1 is modelled as,

$$N_{j,t+1}\sim \text{Poisson}\left(\mathrm{rF}\right),$$

in which $F=\frac{1}{2}{\mathrm{pN}}_{a,t+1}$ and *r* is the mean brood size. Values of *F* were floored to model a natural number of breeding individuals. The effect of density dependence (ψ) on mortality for each individual in the population was then modelled as a function of *N*_*a*,*t*+1_, *N*_*j*,*t*+1_ and a fixed carrying capacity *K*, such that

$$\psi \left(N_{a,t+1},N_{j,t+1},K\right)=\max\left[0,\frac{\left(N_{a,t+1}+N_{j,t+1}\right)-K}{K}\right].$$

Note that if (*N*_*a*,*t*+1_ *+ N*_*j*,*t*+1_) < *K*, then $\psi \left(N_{a,t+1},N_{j,t+1},K\right)=0$ (i.e. all individuals survive if the population size is below carrying capacity), and the probability of mortality increases linearly when (*N*_*a*,*t*+1_ *+ N*_*j*,*t*+1_) > *K*. In addition, values of ψ > 1 were set to 1 (i.e. complete mortality). To model stochastic effects of density dependence applied to each individual, we then model the predicted size of the LWfG population by sampling from a binomial distribution,

$$N_{t+1}\sim \text{Binomial}\left(N_{a,t+1}+N_{j,t+1},1-\psi \left(N_{a,t+1},N_{j,t+1},K\right)\right).$$

All *N*_*t*+1_ individuals in the population become adults at the beginning of the following time step.

We ran simulations for varying levels of illegal offtake (which we interpreted to include accidental offtake by licensed hunters). This was varied from 0 to 10,000 animals (in increments of 100) to represent an extreme scenario in which each hunter within the study area harvested one LWfG. Due to uncertainty related to LWfG return rate, we also varied the value of ϕ between 0.6 and 0.84, reflecting a range of plausible survival values found in populations under reduced harvesting pressure (Lampila, 2001; Schekkerman & Koffijberg, 2019; Schmutz & Ely, 1999). In all simulations, *p* was sampled from a normal distribution with mean 0.5 and standard deviation 0.05 (Cuthbert et al., 2018), *r* set to 1.26 following Cuthbert & Aarvak (2016) and *K* to 250,000 following Mooij (2010). We ran 100 iterations of the 20‐year management period for each combination of illegal offtake and return rate, and assessed population dynamics using three different metrics: goose population growth measured over the entire management period (i.e. [*N*_*t*=20_ − *N*_*t*=0_]/*N*_*t*=0_), mean annual growth rate (i.e. mean{[*N*_*t*+1_ − *N*_*t*_]/*N*_*t*_}), and extinction probability (i.e. number of replicates in which the population reaches an abundance of zero divided by the total number of replicates). Population size at *t* = 0 was set to 32,000 individuals as per the estimated number of individuals in northern Kazakhstan reported by Cuthbert & Aarvak (2016). We tested for the effect of illegal offtake and return rate on measures of population growth and extinction probability using generalised additive models (GAMs, using Gaussian and binomial error structures for growth and extinction probability respectively; r package ‘mgcv’; Wood, 2011). All analyses were carried out in r (R Core Team, 2020).

## 3 RESULTS

We approached 197 people across 46 sites in northern Kazakhstan (Figure 1). Eight individuals declined to participate. Full surveys were completed by 166 men: 42% (*n* = 70) owned hunting licences, and of these licences, a combined ‘Goose and Duck’ licence was most common (*n* = 55; Table S2). Across all licence types, 61 licences (87% of all licences owned) included geese: three goose species (Greylag Goose, Greater White‐fronted Goose and Bean Goose) were listed, alongside ten other waterfowl species (Figure S1). Surveys were completed by 23 women, none of whom hunted.

### 3.1 Motivations for waterfowl hunting

Eighty respondents were in the UCT ‘Control’ and 109 in the ‘Treatment’ groups. Hunting licence ownership was associated with goose hunting activity over a 12‐month period (Table 1c), and goose hunting during the legal hunting season (autumn/winter, Table 1d). Lower levels of hunting during the autumn/winter were associated with households with higher levels of full‐time employment (Table 1d). There was no evidence of engagement in illegal goose hunting during spring/summer (Table 1e), indicating compliance with the newly implemented spring hunting ban. There was no evidence of goose hunting being undertaken for cash, indicating that hunting is not financially motivated (Table 1f).

**TABLE 1:** Fixed‐effect coefficient estimates from generalised linear mixed effects models for (a) correct wildfowl protection status knowledge, (b) correct LWfG protection status knowledge, (c) number of activities in the past year, (d) number of activities in the past autumn/winter, (e) number of activities in the past spring/summer, and (f) number of activities for cash. For models (c)–(f), only interaction terms are presented here (all models included their constituent main effects, but for interpretation purposes only the size and direction of the interaction terms are relevant); full model estimates for these models are presented in Table S4. Estimates for both the final model (i.e. a model including all terms retained in the top ΔAIC < 4 set), as well as model averaged parameter estimates over the ΔAIC < 4 set are presented. Upper and lower bounds are calculated from 1,000 bootstrap samples from fitted models; for averaged models these are summarised across all models within the top set. Models for ‘knowledge’ (a and b) were fitted with Poisson error distributions with log‐link, models for activities (c–f) were fitted with Gaussian distributions. All models included a single random effect for ‘site’. Model selection tables for all these models are presented in Table S3

	Final			Averaged		
	Estimate	Lower 95%Q.	Upper 95%Q.	Estimate	Lower 95%Q.	Upper 95%Q.
(a) Correct WF protection knowledge						
(Intercept)	0.770	0.192	1.354	0.906	0.602	1.177
Age	−0.001	−0.012	0.009	−0.001	−0.009	0.007
Yrs. edu.	0.008	−0.028	0.043	0.006	−0.027	0.038
Mths. empl.	−0.001	−0.031	0.032	−0.004	−0.027	0.023
No. ppl. Empl.	−0.044	−0.131	0.039	−0.028	−0.096	0.038
Yrs. vill.	0.005	−0.007	0.016	0.002	−0.007	0.011
Single licence	0.219	−0.164	0.548	0.216	−0.133	0.538
G&D licence	0.223	−0.028	0.473	0.220	−0.024	0.467
**WF protection knowledge**	**0.258**	**0.003**	**0.525**	**0.315**	**0.077**	**0.566**
(b) Correct LWFG protection knowledge						
(Intercept)	−6.492	−14.146	−2.642	−5.604	−11.424	−3.095
Age	−0.019	−0.097	0.046	−0.005	−0.055	0.040
Yrs. edu.	0.072	−0.170	0.390	0.062	−0.163	0.335
Mths. empl.	−0.017	−0.227	0.164	0.005	−0.161	0.161
Yrs. vill.	0.022	−0.050	0.109	0.003	−0.048	0.057
**No. ppl. Empl.**	**0.678**	**0.169**	**1.546**	**0.557**	**0.221**	**1.250**
Single licence	2.495	−0.235	7.692	1.394	−0.415	31.106
**G&D licence**	**1.474**	**0.023**	**3.180**	1.196	−0.031	2.825
**WF protection knowledge**	**1.535**	**0.673**	**2.865**	**1.506**	**0.854**	**2.990**
(c) Number of activities in past year						
T × LWFG protect knowledge	0.592	−0.151	1.320	0.444	−0.314	1.205
T × Single licence	0.007	−0.970	1.097	0.135	−0.892	1.123
**T × G&D licence**	**0.911**	**0.217**	**1.619**	**0.820**	**0.091**	**1.536**
**T × No. ppl. Empl.**	−0.229	−0.487	0.013	**−0.213**	**−0.395**	**−0.026**
T × WF protect knowledge	−0.307	−0.697	0.080	−0.094	−0.466	0.283
T × Yrs. edu.	0.088	−0.014	0.184	0.064	−0.038	0.167
T × Mths. empl.	−0.052	−0.131	0.032	−0.070	−0.147	0.006
T × Age	0.004	−0.023	0.032	−0.015	−0.035	0.005
(d) Number of activities in autumn/winter						
T × Single licence	−0.052	−0.999	0.976	0.015	−0.960	0.981
**T × G&D licence**	**0.998**	**0.323**	**1.687**	**0.955**	**0.277**	**1.618**
T × mnths. Empl.	−0.060	−0.133	0.014	−0.067	−0.138	0.003
T × WF protection knowledge	0.078	−0.251	0.397	0.167	−0.157	0.510
T × Yrs. edu	0.021	−0.078	0.115	0.001	−0.100	0.098
**T × No. ppl. Empl.**	−0.172	−0.352	0.006	**−0.182**	**−0.359**	**−0.003**
(e) Number of activities in spring/summer						
T × Yrs. edu.	0.069	−0.023	0.176	0.043	−0.050	0.137
T × WF protection knowledge	0.030	−0.304	0.372	0.047	−0.255	0.364
T × Mths. empl.	−0.054	−0.128	0.016	−0.032	−0.102	0.034
T × Yrs. village	−0.004	−0.030	0.021	0.001	−0.025	0.026
(f) Number of activities for cash						
T × Mths. empl.	0.022	−0.034	0.087	0.014	−0.035	0.064
T × yrs. village	−0.009	−0.034	0.016	−0.015	−0.032	0.003
T × No. ppl. Empl.	−0.150	−0.333	0.018	−0.083	−0.213	0.049
T × LWFG protection knowledge	0.170	−0.334	0.701	0.254	−0.184	0.681
T × Yrs. edu.	0.020	−0.047	0.089	0.009	−0.059	0.075
T × WF protection knowledge	0.082	−0.189	0.351	0.156	−0.068	0.373
T × Age	0.008	−0.012	0.028	0.005	−0.015	0.024

### 3.2 Species protection knowledge

**FIGURE 2:**
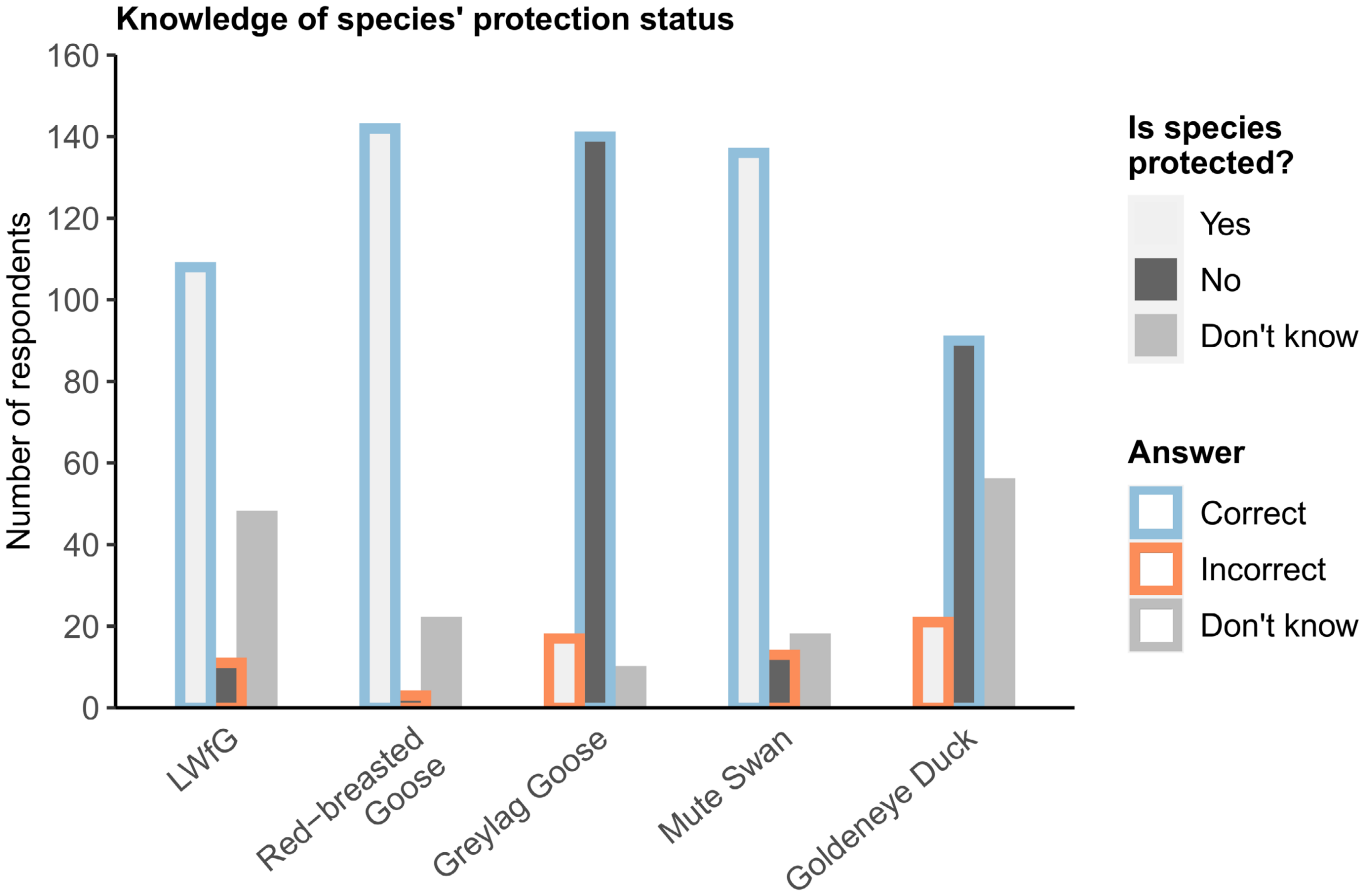
Male respondents' knowledge of species' hunting protection status. LWfG, Red‐breasted Geese, and Mute Swans are protected. Greylag Geese and Goldeneye Ducks are not protected. See Figure S3 for female respondents' knowledge of species protection

We found knowledge of waterfowl species protection status was lacking for both men and women. Focussing on male respondents (as hunting was exclusively undertaken by men in our survey) and our focal species, 6.6% misclassified LWfG as unprotected, while 28.3% stated that they did not know (Figure 2). Eight licensed hunters (11% of hunters licensed to hunt geese) stated that they did not know LWfG are protected (Figure S2). Although those respondents familiar with LWFG protection status also had better knowledge of wildfowl protection status, none of the other predictors were associated with general wildfowl protection status (Table 1a). Hunting licence ownership tended to increase the likelihood of a respondent knowing that LWfG are protected (Table 1b), although this effect overlapped zero based on model averaged estimates. When respondents had greater knowledge of other waterfowl species' protection status, they were more likely to know LWfG are protected (Table 1b). Knowledge of LWfG protection status increased with higher levels of employment in households (Table 1). Of the female respondents (*n* = 23; none of whom hunted) 73.91% did not know LWfG are protected, 8.7% misclassified LWfG as unprotected and 17.39% correctly stated that LWfG are protected (Figure S3).

### 3.3 LWfG population modelling

**FIGURE 3:**
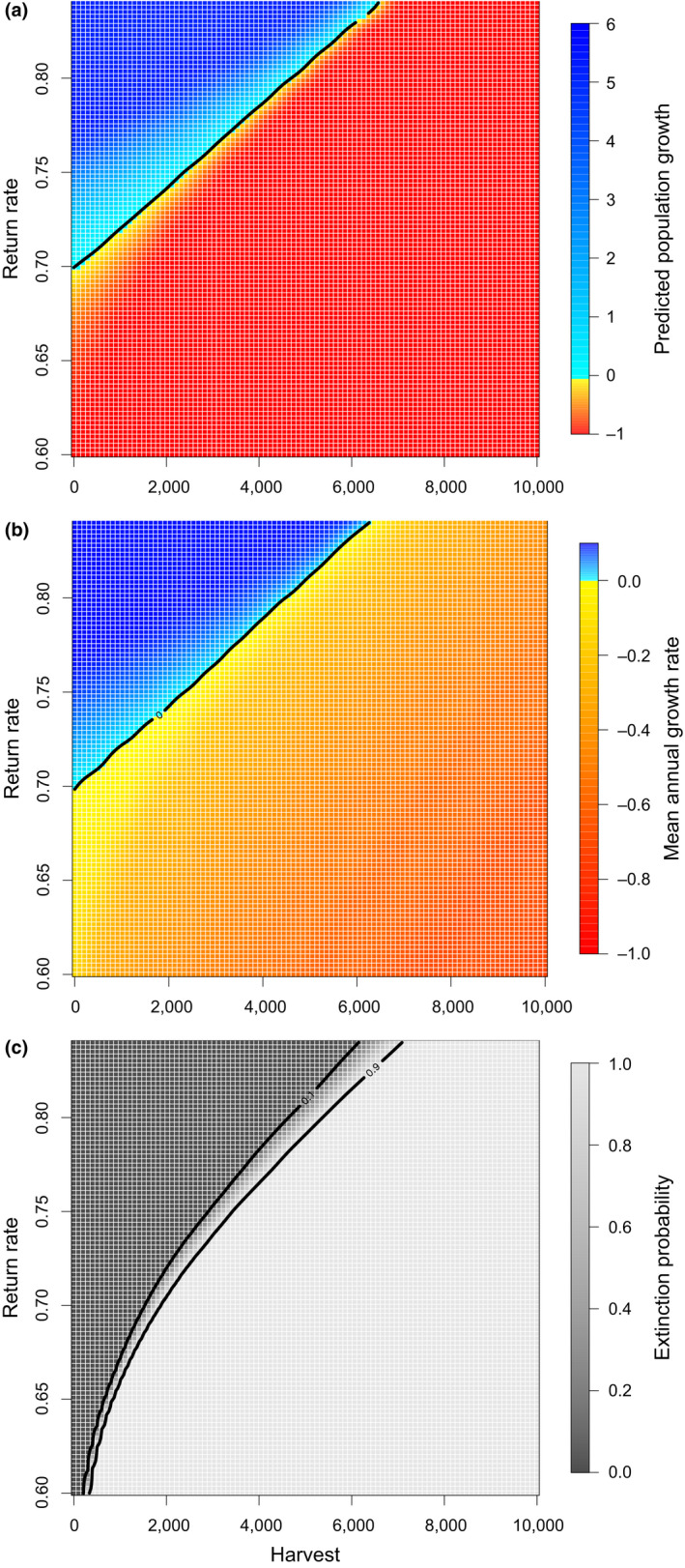
LWfG population growth (a), mean annual growth rate (b), and extinction probability (c) over a 20‐year period as a function of illegal offtake and return rate. Prediction surfaces were obtained from generalised additive models with Gaussian and binomial error structures, respectively (see main text). The contour line in (a) and (b) denotes a population growth rate of 0 (i.e. a stable population), whilst contour lines in (c) reflect extinction probabilities of 0.1 and 0.9

In the absence of illegal offtake, our LWfG population model predicted an average population growth over the 20‐year management period ranging from −1.0 for a return rate of ϕ = 0.6 to 5.3 for ϕ = 0.84 (Figure 3a). Population growth declined steeply as annual harvest level increased and return rate decreased, switching from positive to negative for any offtake value when ϕ < 0.7. Above this return rate threshold, the direction of population growth depended on harvesting pressure, until an offtake level of ~6622 above which population growth was negative regardless of return rate. A similar pattern was found for mean annual growth rate, which ranged from −0.72 for scenarios that combined low return rate and high harvest to 0.10 for scenarios in which return rate was highest and harvest non‐existent (Figure 3b). Lastly, extinction probability shifted rapidly from 0 to 1 as annual harvest level increased and return rate decreased (Figure 3c). In particular, extinction probability was 1 for harvest levels above ~442 and ~7,211 when ϕ = 0.6 and 0.84, respectively. Harvesting threshold for population growth and extinction probability were not sensitive to the value of *K* (Figure S4).

## 4 DISCUSSION

The success of global conservation policies relies on cooperation with and engagement of stakeholders, but there is often a mismatch between global objectives and local actions. Here, we illustrate this point using an internationally protected migratory species, the Lesser White‐fronted Goose (LWfG) passing through staging grounds in northern Kazakhstan, an area popular for recreational hunting. We show that hunting waterfowl in northern Kazakhstan is more strongly associated with social and cultural factors, rather than being financially motivated. We found no evidence that hunters surveyed were non‐compliant with newly implemented seasonal hunting bans and licensing regulations. However, significant knowledge gaps existed surrounding the protection status of waterfowl, including LWfG. Accidental and illegal LWfG offtake occurs, although the exact extent remains unknown. To assess the potential consequences of this, using simulations of population dynamics under varying harvesting scenarios, we showed that the LWfG population is sensitive to changes in the level of potential offtake occurring in northern Kazakhstan, particularly so when survival along the rest of the flyway, here represented by the return rate, was low. More generally, for a given return rate, our predictions suggest the existence of a clear tipping point at which increases in harvest bring about a rapid shift from positive to negative population growth rate values. Taken together, our results highlight the importance of integrating local‐level context and management with conservation policy targeted at the broader migratory range (Bauer et al., 2018).

### 4.1 Importance of local socio‐ecological contexts in achieving global‐level conservation targets

Through conservation interventions under UN‐AEWA, some local stakeholder engagement regarding waterfowl hunting has already occurred in northern Kazakhstan, particularly for red listed species including LWfG: activities have included distributing posters to hunting clubs outlining how to distinguish LWfG within mixed flocks. Nevertheless, we identify significant knowledge gaps relating to LWfG protection status among hunters licensed to hunt geese. Our findings indicate that ~1,100 licensed hunters may have insufficient knowledge of LWfG protection status and may be unknowingly illegally killing LWfG.

In northern Kazakhstan accidental offtake of LWfG due to misidentification has been estimated at one to three LWfG per 100 Greater White‐fronted or Greylag Geese (which can be hunted legally); although in one area offtake was as high as one LWfG per 20–30 Greater White‐fronted or Greylag Geese (Yerokhov, 2013). The 2017 goose hunting offtake limit for northern Kazakhstan (Kostanay Region and North Kazakhstan combined) was 71,240 (ACBK, unpublished data). Combining this offtake limit with estimates of accidental offtake (Yerokhov, 2013), there is therefore the potential for ~700 (up to 3,500 in the extreme) LWfG being accidentally killed per year across the region. According to our population trend scenarios, an offtake level of ~700 could cause a negative population growth rate, and even extinction, when combined with a return rate lower than 0.71. Although up to date, empirical survival estimates for LWfG are sparse, recent estimates for adult survival range from 0.57 to 0.82, with first‐year survival typically lower (Schekkerman & Koffijberg, 2019) further highlighting the potential risk to the population. Thus, it is clear that to achieve global conservation objectives for LWfG, a better understanding of mechanisms driving non‐compliance with hunting regulations (either accidental or deliberate) is vital.

We found no evidence for non‐compliance with the new spring hunting ban, yet hunting was socially and culturally important (von Essen et al., 2019). Indeed, our informal discussions with hunters revealed that some former hunters now choose not to hunt at all, which was attributed to underlying anger that both licence fees had increased and hunting quotas decreased, following efforts to benefit conservation (Jones et al., 2017). Thus, top‐down decisions on appropriate conservation interventions (e.g. hunting bans and quotas) potentially risk disengagement of local stakeholders. It is widely recognised that top‐down approaches alone without closer understanding of stakeholder behaviour limits conservation efficacy both in general (e.g. Milner‐Gulland, 2012) and specifically in management of illegal hunting (Challender & MacMillan, 2014). Managing conflicts ‘at a distance’ may be beneficial to conservation objectives in the short‐term, but detrimental to long‐term sustainability if policies are culturally insensitive, socially inappropriate or irrelevant to local contexts (Ottolini et al., 2020).

The numerous wetlands and lakes and low human population density of northern Kazakhstan offers huge scope for illegal hunting to remain undetected. Hunting inspectors explained that illegal hunters can be wealthy individuals and often ‘outsiders’ from larger towns and cities, with powerful off‐road vehicles that enable easy evasion of inspectors. Despite considerable efforts, limited resources can preclude effective local‐level enforcement of global‐level hunting regulations. In order to bolster local stakeholder engagement, maximise on resources, and embed the social and cultural importance of hunting for local people within conservation policies, we suggest that greater consideration of the knowledge, views and perceptions of hunters can help inform the active management of waterfowl species, similar to goose hunting and adaptive management governance in Denmark (Williams et al., 2019). Hunters in northern Kazakhstan have a keen sense of stewardship of waterfowl populations in order to preserve their ability to hunt. Thus, through effective engagement, for example ensuring that LWfG identification and species protection status is known and accidental offtake is reported without fear of recrimination, resources for enforcement may be focused on deterring illegal hunting. Moreover, engagement targeting increasing women's knowledge of species protection may lead to further positive outcomes regarding hunting behaviour (Espinosa, 2010). Particular focus should be paid to strengthening local institutions and concentrating on who disseminates information to ensure its legitimacy, so that local engagement is more locally and culturally grounded. Moreover, discussion is needed regarding how linkages between local, national and international governance may be further strengthened.

### 4.2 Connecting global policies with varying local socio‐ecological contexts

In order to connect local action and global policy, partnerships of governments, businesses, non‐governmental organisations and local people are crucial (Berkes, 2007). For instance in the context of northern Kazakhstan and conserving LWfG, intergovernmental and NGO partnerships are formed under UN‐AEWA. More widely, the Intergovernmental Science‐Policy Platform on Biodiversity and Ecosystem Services (IPBES) connects science with policy and supports evidence‐informed policy (Doherty et al., 2018). However, our study suggests that a greater role for local people and the incorporation of their knowledge and values as part of equitable decision‐making, would allow for processes towards the mitigation of conflicting objectives of biodiversity conservation versus local culture.

In the case of northern Kazakhstan and LWfG, despite local implementation of hunting bans and restrictions the LWfG population is still declining. Our current knowledge of LWfG population movement, behaviour and demographics is incomplete: drivers of decline may lie in multiple locations along its flyway, despite being covered by international agreements provided by UN‐AEWA (Jones et al., 2008). Indeed, UN‐AEWA covers 255 species of migratory birds, all of which cross international borders and overexploitation is a serious threat (UN‐AEWA, 2018). Our study highlights the need to integrate global‐level policies aimed at species conservation and multidimensional local‐level social, environmental and governance factors: these concepts are applicable and relevant to many different contexts, not least the 255 migratory species covered by UN‐AEWA (Berkes, 2007; Cusack et al., 2020; UN‐AEWA, 2018). Indeed, this approach has been echoed in other contexts of managing migratory bird populations and hunting e.g. Malta (Verissimo & Campbell, 2015) and across e.g. the East Asian‐Australasian Flyway, encompassing different socio‐ecological and governance contexts (Gallo‐Cajiao et al., 2020; Szabo et al., 2016).

### 4.3 Synthesis and policy recommendations

Global conservation policies and targets may not fully address population declines in wide‐ranging or migratory species if interventions are not dynamic and locally appropriate for differing socio‐economic, environmental and governance contexts across range states. In order to address this issue, detailed and long‐term information on socio‐economic, environmental and governance contexts is needed. To gain such information, we recommend the following, complementary, approaches. First, socio‐ecological data, stakeholder decision‐making and species population modelling needs to be fully integrated (c.f. Duthie et al., 2018). This would enable understanding of hotspots of impact across species' ranges, where regions or countries have different resources available for enforcement, and where rates of legal and illegal hunting are higher or lower (Cusack et al., 2020). Crucial to this is the monitoring of migration patterns and return rate across the species' range, information that will enable more realistic and robust population models to be implemented (Bauer et al., 2018). Second, local practices should be more strongly linked with global targets through peer‐to‐peer technology and information sharing (Tinch et al., 2018). This could include, for example, an international platform mobile app building on the extreme citizen science idea (Fritz et al., 2019; Pejovic & Skarlatidou, 2020) where data on hunting bags and population status can be shared (top‐down) alongside photos and hunting reports (bottom‐up) along the entire flyway. Third, taking lessons from the two‐way interaction between the local socio‐ecological context and conservation targets in northern Kazakhstan, we recommend that local contexts should be explicitly integrated with global policies to promote equitable and sustainable management of natural resources, particularly of wide‐ranging or migratory species crossing international borders where socio‐ecological contexts may differ. Such an approach is adaptable and applicable to many different contexts, including, but not limited to, the 255 migratory species covered by UN‐AEWA international agreements.

## AUTHORS' CONTRIBUTIONS

I.L.J., R.C.W., N.B., A.T., I.Z. and K.Z. designed the socio‐ecological surveys; I.L.J., A.T., I.Z. and K.Z. collected the data; I.L.J., J.J.C. and J.M. led the data analysis. All authors contributed important intellectual content and to manuscript writing and editing; all authors gave final approval for publication.

## Supporting information


**Appendix S1** Supporting Information

## Data Availability

Data available via the University of Stirling dataSTORRE http://hdl.handle.net/11667/195 (Jones et al., 2022).
