# Supplementary material for: Achieving international biodiversity targets: Learning from local norms, values and actions regarding migratory waterfowl management in Kazakhstan
Source: J Appl Ecol. 2022 May 30;59(7):1911–24. doi: 10.1111/1365-2664.14198 (PMC9543466; doi:10.1111/1365-2664.14198)
Supplement: Supplementary file 1 — Appendix S1 Supporting Information [file JPE-59-1911-s001.pdf]

SUPPORTING INFORMATION

Table S1: Summary of variables used in mixed effects modelling. For statistical analyses licences for 'only geese' and 'only ducks' were combined into a 'single licence' group.

Variable type	Predictor variable	Description
Demographic	Age	Continuous (range = 18-77; mean = 45.8; sd = 13.4)
	Years formal education	Continuous (range = 8-30; mean = 13; sd = 3)
	Months employed in past year	Continuous (range = 0-12; mean = 9.3; sd = 4.4)
	Number of people in household in full-time employment	Continuous (range = 0-9; mean = 2; sd = 1.5)
	Years in village	Continuous (range = 1-77; mean = 25.9; sd = 13.7)
Hunting licence	Licence type	Categorical (goose and duck, n = 55; only goose, n = 6; only duck, n = 9; no licence, n = 48)
Knowledge	Score of correct answers regarding species' protection status	Continuous (range = 0-4; mean = 3; sd = 1)
	LWfG protection status	Categorical (correct, n = 108; incorrect, n = 58)

Table S2: Summary of male respondents and hunting licence ownership in treatment and control survey groups. Single licences (goose licence only and other bird licence only) were combined in analyses to give one 'single licence' level within the 'licence type' variable. No female respondents owned a hunting licence.

Survey group	Number of respondents	Goose and duck licence	Goose licence only	Other bird licence only	No licence
Control	64	23	2	4	18
Treatment	102	32	4	5	30

Table S3: Top delta AICc<4 model selection tables for mixed-effects models for (a) correct wildfowl protection status knowledge, (b) correct LWfG protection status knowledge, (c) number of activities in the past year, (d) number of activities in the past autumn/winter, (e) number of activities in the past spring/summer, and (f) number of activities for cash. Models for "knowledge" (a and b) were fitted with Poisson error distributions with log-link, models for activities (c-f) were fitted with Gaussian distributions. All models included a single random effect for "site". A + indicates the inclusion of a factorial predictor.

Table S3. (a) Correct wildfowl protection status knowledge. Full model set included 128 models. Intercept only model ranked 48th with AICc of 402.48. Fully saturated model ranked 111th with AICc of 407.44

	(Intercept)	Age	LWFG protection knowledge	Licence	Mnths. empl.	No. ppl. empl.	Yrs. edu.	Yrs. in village	df	log. Lik.	AICc	Delta	Weight
1	0.916		0.333						3	-195.00	396.22	0.000	0.190
2	0.951		0.360			-0.025			4	-194.71	397.78	1.566	0.087
3	0.860		0.236	+					5	-193.64	397.82	1.605	0.085
4	0.840		0.325				0.006		4	-194.94	398.22	2.007	0.070
5	0.890		0.329					0.001	4	-194.96	398.28	2.065	0.068
6	0.942		0.340		-0.003				4	-194.97	398.29	2.079	0.067
7	0.950	-0.001	0.336						4	-194.98	398.32	2.101	0.066
8	0.965			+					4	-195.27	398.89	2.676	0.050
9	0.900		0.263	+		-0.030			6	-193.23	399.22	3.001	0.042
10	0.899		0.361			-0.034		0.003	5	-194.51	399.56	3.340	0.036
11	0.837		0.352			-0.029	0.010		5	-194.56	399.65	3.435	0.034
12	0.914		0.245	+	-0.007				6	-193.49	399.74	3.521	0.033
13	0.820		0.229	+				0.002	6	-193.56	399.87	3.654	0.031
14	0.917	0.001	0.361			-0.029			5	-194.69	399.92	3.704	0.030
15	0.951		0.360		0.000	-0.025			5	-194.71	399.96	3.748	0.029
16	0.905	-0.001	0.240	+					6	-193.60	399.96	3.748	0.029
17	0.812		0.233	+			0.004		6	-193.62	399.99	3.774	0.029
18	0.959	-0.003	0.333					0.003	5	-194.83	400.20	3.984	0.026

Table S3. (b) Correct LWfG protection knowledge. Full model set included 128 models. Intercept only model ranked 125th with an AICc of 151.55. Fully saturated model ranked 37th with an AICc of 122.42.

	(Intercept)	Age	Licence	Mnths. empl.	No. ppl. empl.	Wildfowl protection knowledge	Yrs. edu.	Yrs. in village	df	log. Lik.	AICc	Delta	Weight
1	-5.051		+		0.524	1.303			6	-50.51	113.78	0.000	0.224
2	-6.063				0.597	1.755			4	-52.85	114.06	0.284	0.194
3	-5.653		+		0.507	1.282	0.055		7	-50.37	115.76	1.989	0.083
4	-6.686				0.578	1.687	0.069		5	-52.66	115.85	2.076	0.079
5	-5.221		+		0.510	1.309		0.007	7	-50.47	115.96	2.181	0.075
6	-4.810	-0.006	+		0.549	1.296			7	-50.48	115.97	2.194	0.075
7	-5.051		+	-0.004	0.529	1.310			7	-50.51	116.03	2.259	0.072
8	-5.842	-0.005			0.620	1.747			5	-52.83	116.19	2.416	0.067
9	-6.049			0.015	0.580	1.720			5	-52.83	116.20	2.425	0.067
10	-6.030				0.599	1.752		-0.001	5	-52.85	116.24	2.464	0.06

Table S3 (c) Number of activities in past year. Full model set included 257 models. Intercept only model ranked 236th with AICc of 315.17, fully saturated model ranked 181st with AICc of 309.19.

	(Intercept	Age	Treatment	LWFG protection knowledge	Licence	Mnths. empl.	No. ppl. empl.	WF protection knowledge	Yrs. edu	Yrs. village	T * Age	protection	T * licence	T * Mnths. empl.	T * No. ppl. empl.	T * WF protection knowledge	T * Yrs. edu.	T * Yrs. village	df	log. Lik.	AICc	Delta	Weight
1	1.846		+	-0.827	+		0.151	0.462	-			+	+		+	+	+		16	-129.05	295.71	0.000	0.127
2	1.188		+	-0.837	+		0.118	0.367				+	+		+	+			14	-131.86	295.96	0.243	0.112
3	1.511		+	-0.668	+			0.304				+	+			+			12	-134.47	296.02	0.307	0.109
4	1.329		+	-0.708	+	0.022		0.324				+	+	+		+			14	-132.25	296.75	1.037	0.075
5	1.901		+	-0.681	+	0.044		0.409	-			+	+	+		+	+		16	-129.83	297.26	1.549	0.058
6	2.101		+	-0.624	+			0.359	-			+	+			+	+		14	-132.65	297.55	1.831	0.051
7	1.971		+		+								+						8	-140.32	298.01	2.297	0.040
8	1.509		+					0.175								+			6	-142.67	298.13	2.416	0.038
9	1.763		+	-0.837	+	0.022	0.134	0.478	-			+	+	+	+	+	+		18	-127.47	298.14	2.425	0.038
10	1.464		+		+			0.184					+			+			10	-138.01	298.15	2.439	0.037
11	1.563		+	-0.657				0.308				+				+			8	-140.46	298.30	2.581	0.035
12	1.900		+		+	0.010							+	+					10	-138.13	298.39	2.673	0.033
13	1.348		+		+	0.012		0.194					+	+		+			12	-135.69	298.47	2.758	0.032
14	1.946		+		+	0.036		0.287	-				+	+		+	+		14	-133.18	298.60	2.884	0.030
15	1.166		+	-0.847	+	0.001	0.116	0.375				+	+	+	+	+			16	-130.51	298.63	2.918	0.029
16	2.113		+		+			0.251	-				+			+	+		12	-135.80	298.69	2.978	0.029
17	1.868		+		+		0.048						+		+				10	-138.37	298.87	3.154	0.026
18	1.978		+		+		0.092	0.292	-				+		+	+	+		14	-133.46	299.15	3.437	0.023
19	1.249	0.007	+	-0.703	+			0.294			+	+	+			+			14	-133.48	299.20	3.488	0.022
20	1.311		+		+		0.057	0.196					+		+	+			12	-136.14	299.37	3.650	0.020
21	1.263		+	-0.826			0.113	0.356				+			+	+			10	-138.70	299.54	3.820	0.019
22	2.658		+		+				-				+				+		10	-138.75	299.63	3.916	0.018

Table S3 (d) Number of activities in autumn/winter. Full model set included 257 models. Intercept only model ranked 257th with AICc of 322.15. Fully saturated model ranked 188th with AICc of 309.53.

	(Intercept	Treatment	Licence	Mnths. empl.	No. ppl. empl.	WF protection knowledge	Yrs. edu	T * licence	T * Mnths. empl.	T * No. ppl. empl.	T * WF protection knowledge	T * Yrs. edu.	df	log. Lik.	AICc	Delta	Weight
1	1.432	+	+	0.007		0.143		+	+		+		12	-132.37	291.75	0.000	0.280
2	1.825	+	+	0.017		0.197	-0.053	+	+		+	+	14	-130.38	292.89	1.140	0.158
3	1.460	+	+	-0.005	0.031	0.141		+	+	+	+		14	-130.50	293.12	1.370	0.141
4	1.697	+		-0.013		0.053			+		+		8	-138.11	293.56	1.812	0.113
5	1.445	+	+		0.032	0.133		+		+	+		12	-133.69	294.38	2.636	0.075
6	1.901	+	+		0.055	0.199	-0.059	+		+	+	+	14	-131.17	294.45	2.706	0.072
7	1.871	+	+	0.006	0.047	0.208	-0.060	+	+	+	+	+	16	-128.51	294.46	2.709	0.072
8	1.716	+		-0.026	0.038	0.051			+	+	+		10	-136.60	295.27	3.527	0.048
9	1.845	+		-0.011		0.073	-0.019		+		+	+	10	-136.75	295.58	3.835	0.041

Table S3 (e) Number of activities in spring/summer. Full model set included 257 models. Fully saturated model ranked 253rd with AICc of 310.8.

	**(Intercept**	**Treatment**	**Mnths. empl.**	**WF protection knowledge**	**Yrs. edu**	**Yrs. village**	**T * Mnths. empl.**	**T * WF protection knowledge**	**T * Yrs. edu.**	**T * Yrs. village**	**df**	**log. Lik.**	**AICc**	**Delta**	**Weight**
1	1.498	+		0.122				+			6	-137.63	288.02	0.000	0.304
2	2.033	+		0.196	-0.058			+	+		8	-135.80	288.92	0.905	0.194
3	1.897	+									4	-140.39	289.14	1.120	0.174
4	1.550	+	-0.008	0.125			+	+			8	-136.64	290.60	2.584	0.084
5	2.076										3	-142.37	290.95	2.933	0.070
6	2.516	+			-0.047				+		6	-139.18	291.12	3.096	0.065
7	1.429	+		0.105		0.006		+		+	8	-137.03	291.37	3.355	0.057
8	1.997	+	0.012	0.199	-0.065		+	+	+		10	-134.73	291.51	3.497	0.053

Table S3 (f) Number of activities for cash. Full model set included 257 models. Fully saturated model ranked 244rd with AICc of 220.77.

	INT	Age	Treatm.	LWFG protection knowledge	Mnths. empl.	No. ppl. empl.	WF protection knowledge	Yrs. edu	Yrs. village	T * Age	T * LWFG protection knowledge	T * Mnths. empl.	T * No. ppl. empl.	T * WF protection knowledge	T * Yrs. edu.	T * Yrs. village	df	log. Lik.	AICc	Delta	Weight
1	0.511		+						-0.001							+	6	-94.71	202.18	0	0.161
2	0.880		+		-0.038				-0.006			+				+	8	-92.53	202.39	0.207	0.145
3	0.928		+					-0.036	0.000						+	+	8	-93.15	202.39	1.443	0.078
4	0.538		+				-0.013		-0.001					+		+	8	-93.21	203.75	1.563	0.073
5	0.981		+		-0.036		-0.043		-0.005			+		+		+	10	-90.85	203.78	1.592	0.072
6	0.491		+			-0.038			0.000				+			+	8	-93.49	204.31	2.130	0.055
7	0.759		+			-0.048	-0.094		0.003				+	+		+	10	-91.22	204.52	2.338	0.050
8	0.919		+		-0.043	0.024			-0.008			+	+			+	10	-91.42	204.91	2.732	0.041
9	0.492		+			-0.038							+				6	-96.09	204.94	2.754	0.040
10	0.765		+			-0.041	-0.083						+	+			8	-93.84	205.02	2.836	0.039
11	0.454																3	-99.44	205.10	2.917	0.037
12	0.652		+	-0.187					-0.001		+					+	8	-93.90	205.13	2.951	0.037
13	1.085		+		-0.027			-0.026	-0.004			+			+	+	10	-91.58	205.24	3.059	0.035
14	1.007		+				-0.028	-0.038	0.000					+	+	+	10	-91.62	205.31	3.130	0.034
15	0.946		+			-0.019		-0.037	0.000				+		+	+	10	-91.73	205.55	3.363	0.030
16	0.830	0.002	+		-0.038				-0.008	+		+				+	10	-91.91	205.89	3.705	0.025
17	0.972		+	-0.184	-0.034				-0.006		+	+				+	10	-91.93	205.94	3.758	0.025
18	0.983		+		-0.040	0.016	-0.032		-0.006			+	+	+		+	12	-89.51	206.03	3.844	0.023

Table S4: Parameter estimates and bootstrapped confidence intervals for GLMMs for UCT data analysis; (a) number of activities in the past year, (b) number of activities in the autumn/winter, (c) number of activities in the spring/summer, (d) number of activities for cash, here also including main effects for each model, but otherwise the same as Table 1 in the main text.

Table S4 (a) Number of activities in the last year.

	**Final**			**Averaged**		
	**Estimate**	**Lower 95%Q.**	**Upper 95%Q.**	**Estimate**	**Lower 95%Q.**	**Upper 95%Q.**
(Intercept)	1.975	0.622	3.360	1.638	0.694	2.597
Treatment	0.509	-1.156	2.349	0.440	-0.778	1.652
LWFG protect knowledge	-0.856	-1.423	-0.275	-0.750	-1.328	-0.171
Single licence	0.693	-0.111	1.466	0.477	-0.303	1.275
Goose & Duck licence	-0.032	-0.557	0.526	0.012	-0.545	0.591
No. ppl. empl.	0.191	-0.008	0.380	0.120	-0.011	0.255
WF protect knowledge	0.529	0.197	0.861	0.338	0.021	0.649
Yrs. edu.	-0.091	-0.159	-0.017	-0.079	-0.152	-0.009
Mths. empl.	0.012	-0.055	0.081	0.023	-0.036	0.085
Age	-0.009	-0.033	0.016	0.007	-0.010	0.024
T * LWFG protect knowledge	0.592	-0.151	1.320	0.444	-0.314	1.205
T * Single licence	0.007	-0.970	1.097	0.135	-0.892	1.123
T * G&D licence	0.911	0.217	1.619	0.820	0.091	1.536
T * No. ppl. empl.	-0.229	-0.487	0.013	-0.213	-0.395	-0.026
T * WF protect knowledge	-0.307	-0.697	0.080	-0.094	-0.466	0.283
T * Yrs. edu.	0.088	-0.014	0.184	0.064	-0.038	0.167
T * Mths. empl.	-0.052	-0.131	0.032	-0.070	-0.147	0.006
T * Age	0.004	-0.023	0.032	-0.015	-0.035	0.005

Table S4 (b) Number of activities in the autumn/winter.

	**Final**			**Averaged**		
	**Estimate**	**Lower 95%Q.**	**Upper 95%Q.**	**Estimate**	**Lower 95%Q.**	**Upper 95%Q.**
(Intercept)	1.888	0.877	2.910	1.625	0.664	2.640
Treatment	0.697	-0.712	1.971	0.567	-0.681	1.783
Single licence	0.476	-0.312	1.227	0.377	-0.370	1.132
G&D licence	-0.451	-0.997	0.096	-0.431	-0.957	0.099
Mnths. empl.	0.006	-0.058	0.066	0.001	-0.054	0.056
No. ppl. empl.	0.048	-0.076	0.179	0.039	-0.082	0.160
WF protection knowledge	0.206	-0.063	0.480	0.142	-0.146	0.412
Yrs. edu.	-0.061	-0.124	0.004	-0.052	-0.119	0.018
T * Single licence	-0.052	-0.999	0.976	0.015	-0.960	0.981
T * G&D licence	0.998	0.323	1.687	0.955	0.277	1.618
T * mnths. empl.	-0.060	-0.133	0.014	-0.067	-0.138	0.003
T * WF protection knowledge	0.078	-0.251	0.397	0.167	-0.157	0.510
T * Yrs. edu	0.021	-0.078	0.115	0.001	-0.100	0.098
T * No. ppl. empl.	-0.172	-0.352	0.006	-0.182	-0.359	-0.003

Table S4(c) Number of activities in the spring/summer.

	**Final**			**Averaged**		
	**Estimate**	**Lower 95%Q.**	**Upper 95%Q.**	**Estimate**	**Lower 95%Q.**	**Upper 95%Q.**
(Intercept	1.850	0.864	2.867	1.804	1.065	2.557
Treatment	-0.124	-1.648	1.430	0.056	-0.996	1.069
Yrs. edu.	-0.068	-0.139	-0.002	-0.057	-0.118	0.003
WF protection knowledge	0.168	-0.125	0.440	0.148	-0.120	0.402
Mths. empl.	0.022	-0.038	0.079	0.000	-0.051	0.052
Yrs. village	0.009	-0.012	0.029	0.006	-0.014	0.025
T * Yrs. edu.	0.069	-0.023	0.176	0.043	-0.050	0.137
T * WF protection knowledge	0.030	-0.304	0.372	0.047	-0.255	0.364
T * Mths. empl.	-0.054	-0.128	0.016	-0.032	-0.102	0.034
T * Yrs. village	-0.004	-0.030	0.021	0.001	-0.025	0.026

Table S4 (d) Number of activities for cash.

	Final			Averaged		
	**Estimate**	**Lower 95%Q.**	**Upper 95%Q.**	**Estimate**	**Lower 95%Q.**	**Upper 95%Q.**
(Intercept	1.096	0.250	1.908	0.753	0.233	1.262
Treatment	-0.692	-1.967	0.548	0.086	-0.611	0.790
Mnths empl.	-0.039	-0.093	0.011	-0.037	-0.075	0.002
Yrs. village	-0.011	-0.033	0.008	-0.003	-0.017	0.011
No. ppl. empl.	0.056	-0.078	0.204	-0.025	-0.118	0.068
WF protection knowledge	0.070	-0.165	0.301	-0.047	-0.233	0.140
Yrs. edu.	-0.024	-0.069	0.018	-0.035	-0.077	0.006
LWFG protection knowledge	-0.266	-0.659	0.134	-0.185	-0.513	0.140
Age	0.002	-0.014	0.018	0.002	-0.013	0.017
T * Mths. empl.	0.022	-0.034	0.087	0.014	-0.035	0.064
T * yrs. village	-0.009	-0.034	0.016	-0.015	-0.032	0.003
T * No. ppl. empl.	-0.150	-0.333	0.018	-0.083	-0.213	0.049
T * LWFG protection knowledge	0.170	-0.334	0.701	0.254	-0.184	0.681
T * Yrs. edu.	0.020	-0.047	0.089	0.009	-0.059	0.075
T * WF protection knowledge	0.082	-0.189	0.351	0.156	-0.068	0.373
T * Age	0.008	-0.012	0.028	0.005	-0.015	0.024

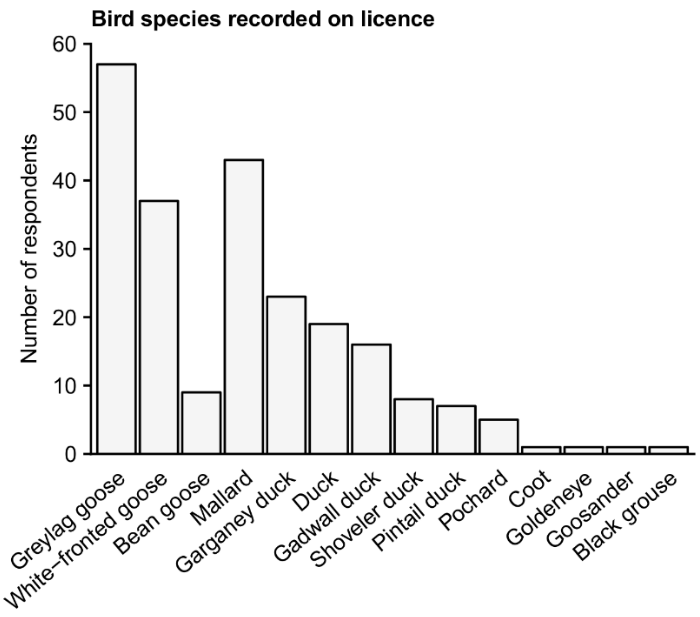

Fig. S1: Species of birds listed on hunting licences owned by survey respondents.

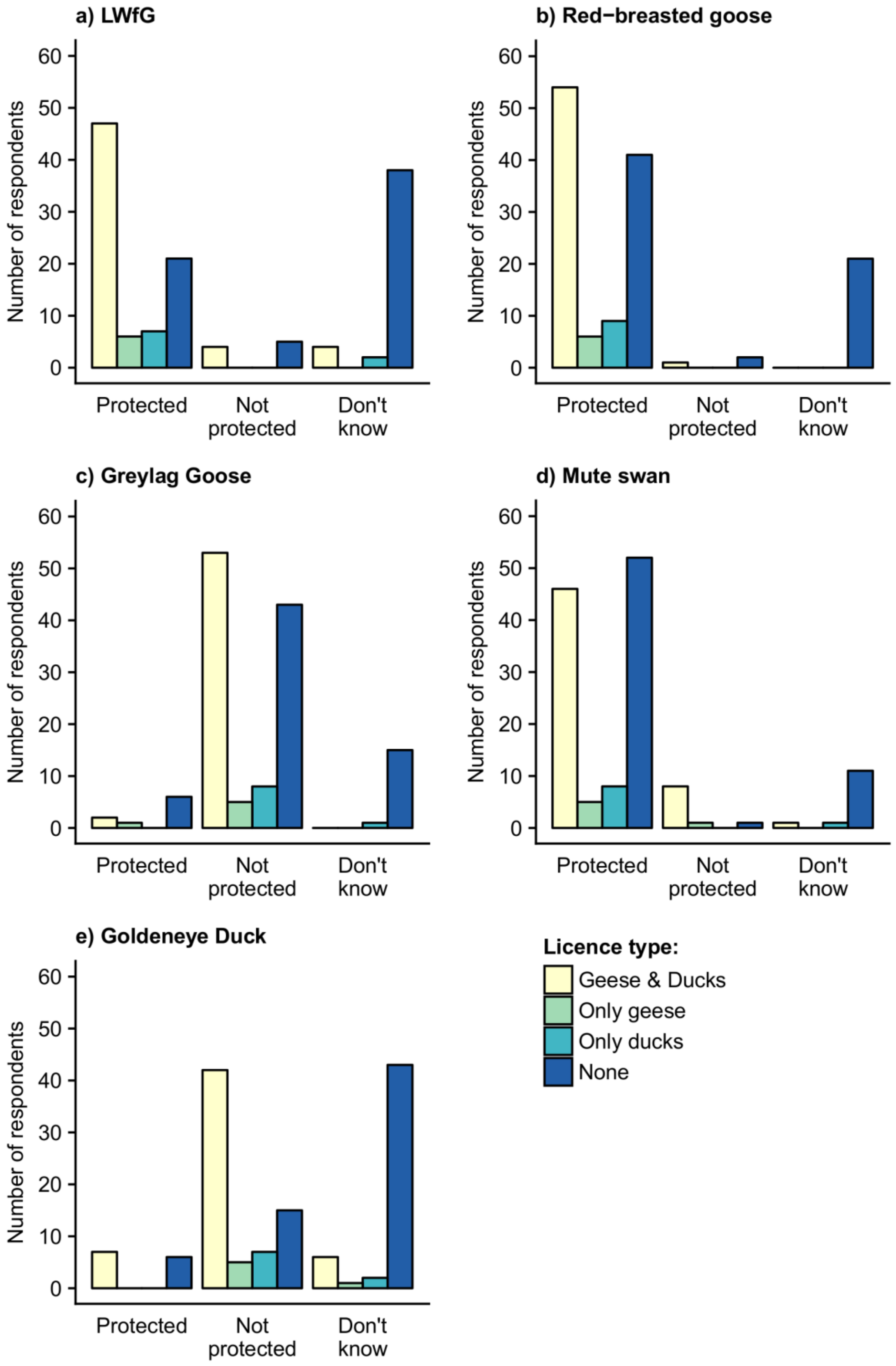

Fig. S2: Summary of knowledge of species' protection status depending on the type of hunting licence owned. LWfG, red breasted geese, and mute swans are protected from hunting. Greylag geese and Goldeneye ducks are not protected from hunting. Eight respondents with a licence to hunt geese (11 % of respondents with a goose hunting licence) did not know LWfG were protected.

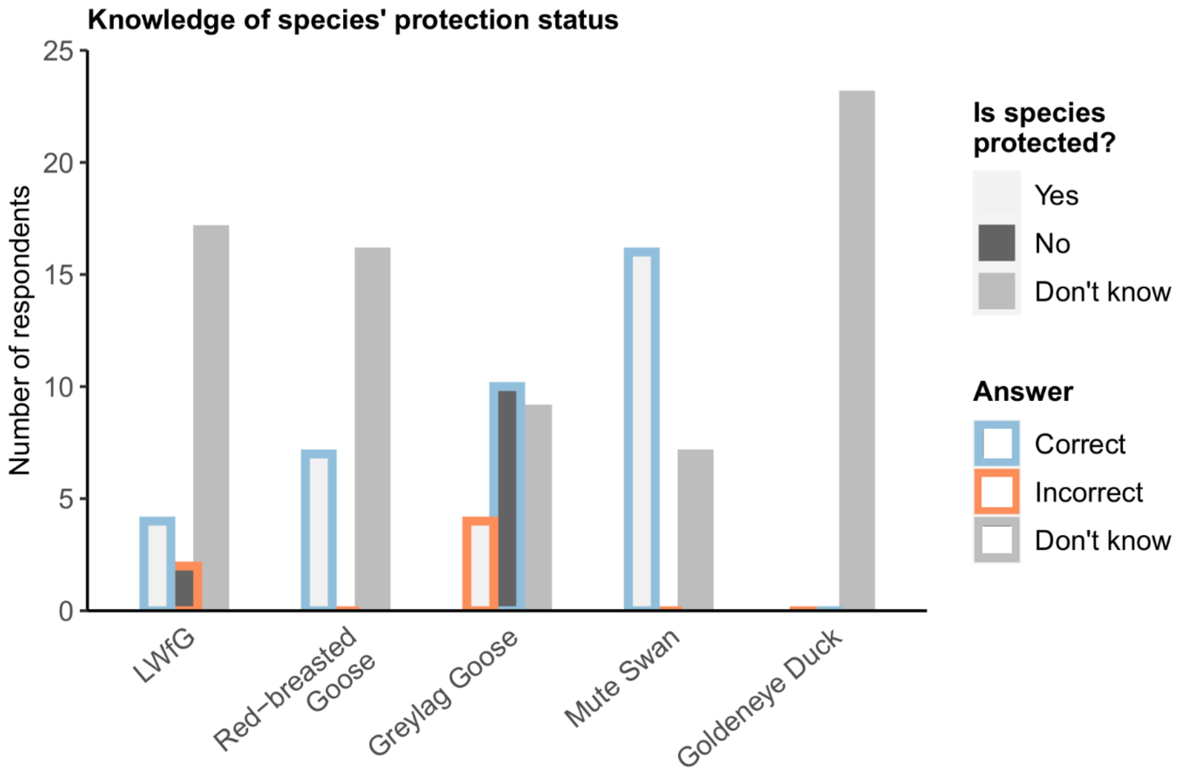

Fig. S3: Summary of female respondents' knowledge of species protection status. LWfG, Red-breasted Geese, and Mute Swans are protected from hunting. Greylag Geese and Goldeneye Ducks are not protected from hunting. No females surveyed owned a hunting licence or participated in hunting (at the time of writing there is one registered female hunter in N Kazakhstan).

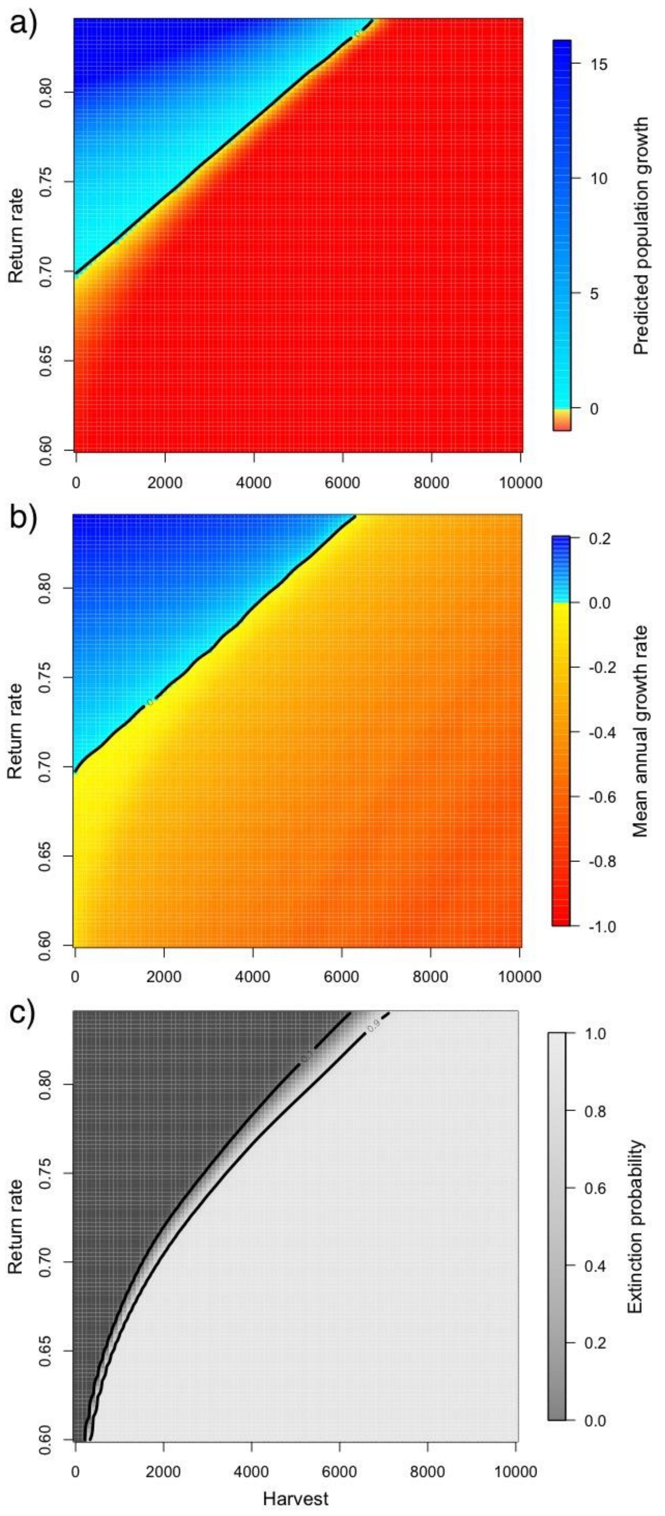

Fig. S4: LWfG population growth (a), mean annual growth rate (b), and extinction probability (c) over a 20-year period as a function of illegal offtake and return rate, in the absence of a fixed carrying capacity. Prediction surfaces were obtained from generalised additive models with Gaussian and Binomial error structures, respectively (see main text). The contour line in (a) and (b) denotes a population growth rate of 0 (i.e. a stable population), whilst contour lines in (c) reflect extinction probabilities of 0.1 and 0.9.

APPENDIX S1

[Questionnaire format modified from Nuno, Bunnefeld, Naiman and Milner-Gulland (2013). A novel approach to assessing the prevalence and drivers of illegal bushmeat hunting in the Serengeti. Conservation Biology 27(6) 1355-1365]

Анкета: Казахстан 2017 / Questionnaire: Kazakhstan 2017

Interviewer/Интервьюер: ____

Date/Дата: ________________

Village/Село: ___________________________ GPS coordinates _________________

INSTRUCTIONS FOR ENUMERATOR: / ИНСТРУКЦИЯ ДЛЯ УЧЕТЧИКА:

Before starting the questionnaire, you must "play a game" to know which cards should be used for this person or household. Here are the rules: / Перед началом опроса вы должны «сыграть в игру», чтобы узнать, какие карты следует использовать для этого человека или семьи. Правила:

Toss a coin, if you get: / Подбросить монетку, если будет:

HEADS, please use cards **C / ОРЕЛ**, используйте карточки **C**;

TAILS, please use cards **T / РЕШКА**, используйте карточки **T**.

Which side of the coin did you get? / Какая сторона монеты выпала?

____

Which cards will you use? / Какие карточки вы будете использовать?

____

Please, always follow these rules! Thank you!

Пожалуйста, всегда соблюдайте эти правила! Спасибо!

PLEASE READ OUT/: ПОЖАЛУЙСТА, ПРОЧТИТЕ:

"My name is [name of enumerator]. I am here on behalf of The Association for the Conservation of Biodiversity in Kazakhstan and the University of Stirling, who are collecting information for a study about people and wildlife in Kazakhstan. We are conducting a short questionnaire about rural communities in Kazakhstan and this will only take a few minutes.

If you choose to take part in the questionnaire, your name will not be recorded and your answers will not be shared with other members of the community or the authorities. Would you like to continue with the questions?"

"Меня зовут [имя учетчика]. Я здесь от имени Ассоциации сохранения биоразнообразия в Казахстане и Университета Стирлинга, которые собирают информацию для изучения людей и дикой природы в Казахстане. Мы проводим короткий опросник о сельских сообществах в Казахстане, и это займет всего несколько минут.

Если вы решите принять участие в анкете, ваше имя не будет записано, и ваши ответы не будут переданы другим членам сообщества или властям. Вы хотели бы продолжить анкетирование?"

[If **NO**, write gender and approximate age of respondent and FINISH HERE]

[Если НЕТ, напишите пол и приблизительный возраст респондента и ЗАВЕРШИТЕ ЗДЕСЬ]

Gender/Пол: Male/Мужской _____ Female/Женский _____

Age/Возраст: 16-25 ______ 26-45 _____ 46-65 _____ 66+ _____

[If **YES**, write down time interview started / Если ДА, записать время начала]

Start time/ Время начала: ___________

Section A: Individual socio-demographic information (about respondent only)

Раздел A: Индивидуальная социально-демографическая информация

A1. Gender/Пол: Male/Мужской ______ Female/Женский _______

A2. Age/Возраст: ____________

A3. Are you the head of household?/ Вы – глава семьи? Yes/Да ____No/Нет ____

A4. Ethnic group/Национальность [Circle one/ Выберите одну]

a) Kazakh / Казах

b) Russian / Русский

c) Ukrainian / Украинец

d) German / Немец

e) Other / Другая __________________________

A5. How many years have you spent in formal education?/ Сколько лет вы получали формальное образование? __________________

A6. In the last year (between September 2016 and August 2017) how many months have you been employed?/ За последний год (с Сентября 2016 по Август 2017) сколько месяцев вы были трудоустроены? ___________________________

Section B: Household socio-demographic information (about household)

Раздел B: Семейная социально-демографическая информация (о семье)

B1. In your household:/ В вашей семье:

a) How many adult males (18 years old or older) are there? / Сколько взрослых мужчин (старше 18 лет)? _______________

b) How many adult females (18 years old or older) are there? / Сколько взрослых женщин (старше 18 лет)? _____________

c) How many children (younger than 18 years old) are there? / Сколько детей (младше 18 лет)? _____________

B2. During the last year (between September 2016 and September 2017), how many people in your household had:/ В течение последнего года (с Сентября 2016 по Сентябрь 2017), сколько людей в вашей семье имели:

a) Full-time employment?/ Полную занятость? _______________

b) Seasonal employment?/ Частичную занятость? ______________

c) No employment?/ Безработные? ____________________

B3. How many years has your household lived in this village? / Сколько лет Ваша семья живет в этом селе? _______________

<u>Section C: CONTROL</u> <u>(ASK ONLY IF YOU GOT **HEADS** AT THE START)</u>

<u>Раздел C: Контроль</u> <u>(СПРАШИВАТЬ ТОЛЬКО ЕСЛИ ВЫПАЛ ОРЕЛ)</u>

[Read out/ Прочтите] *I am going to show you a card with activities. I am going to read their names and then I want you to tell me <u>how many</u> of these activities you have done. Please, <u>don't tell me which ones, just tell me HOW MANY.</u>*

Я покажу вам карточку с действиями. Я собираюсь прочитать их названия, а затем я хочу, чтобы вы рассказали мне, сколько из этих действий вы выполняли. Пожалуйста, не говорите мне, какие именно, просто скажите мне, СКОЛЬКО

[Show card **C Training**, read the names and ask if the person understands/ Показываете карточку С, читаете названия и спрашиваете респондента]

C1. How many of these activities have you done *in the past month*?/ Сколько из этих действий вы сделали за последний месяц? [Circle answer/ Обведите ответ]

1 2 3 4

[Read out/ Прочтите] *Thank you, I am going to show you a card with some activities again. I am going to read their names, and then I want you to tell me <u>how many</u> of these activities you have done.*

Please, <u>don't tell me which ones, just tell me HOW MANY.</u>

Спасибо, Я снова собираюсь показать вам карточку с действиями. Я собираюсь прочитать их названия, а затем я хочу, чтобы вы рассказали мне, сколько из этих действий вы выполняли. Пожалуйста, не говорите мне, какие именно, просто скажите мне, СКОЛЬКО.

[Show card **C Livelihood activities**, read the names and ask if the person understands/ Показываете карточку С, читаете названия и спрашиваете респондента:]

C2. How many of these activities have you done *in the past year?* (September 2016-September 2017)? / Сколько из этих действий вы выполняли в прошлом году? (Сентябрь 2016- Сентябрь 2017)? [Circle answer/ Обведите ответ]

1 2 3 4

C3. And how many of these activities have you done *in the Autumn/Winter?* (September-February)? / Сколько из этих действий вы выполняли Осенью / Зимой? (Сентябрь-Февраль)? [Circle answer/ Обведите ответ]

1 2 3 4

C4. And how many of these activities have you done *in the Spring/Summer?* (March-August)? / Сколько из этих действий вы выполняли Весной / Летом? (Март-Август)? [Circle answer/ Обведите ответ]

1 2 3 4

C5. And how many of these activities have you done *for cash?* / Сколько из этих действий вы выполняли *за оплату?* [Circle answer/ Обведите ответ]

1 2 3 4

[Read out/ Прочтите]

Thank you, now I am going to read a list of animals, and for each animal I want you to tell me if you think they are a protected species or not. Please just say yes, no, or don't know.

Спасибо, теперь я собираюсь прочитать вам список животных, и я хочу, чтобы вы сказали мне, какие из этих животных относятся к охраняемым видам. Пожалуйста, просто отвечайте «да», «нет» или «не знаю».

C6. Which of these species is a protected species? / Какие из этих видов являются защищенными? [Circle answer/ Обведите ответ]

Mute swan / Лебедь-шипун	Yes/Да	/ No/Нет	/ Don't know/Не знаю
Goldeneye / Гоголь	Yes/Да	/ No/Нет	/ Don't know/Не знаю
Lesser White-fronted goose / Пискулька	Yes/Да	/ No/Нет	/ Don't know/Не знаю
Greylag goose / Серый гусь	Yes/Да	/ No/Нет	/ Don't know/Не знаю
Red-breasted goose / Краснозобая казарка	Yes/Да	/ No/Нет	/ Don't know/Не знаю

[Go to section D/Переходите к разделу D]

Section T: TREATMENT (ASK ONLY IF YOU GOT **TAILS** AT THE START)

Раздел Т: ОБРАБОТКА (ИСПОЛЬЗОВАТЬ, ЕСЛИ ВЫПАЛА РЕШКА)

[Read out/ Прочтите]

I am going to show you a card with activities. I am going to read their names and then I want you to tell me how many of these activities you have done.

Please, don't tell me which ones, just tell me HOW MANY.

Я собираюсь показать вам карточку с действиями. Я собираюсь прочитать их названия, а затем я хочу, чтобы вы рассказали мне, сколько из этих действий вы выполняли. Пожалуйста, не говорите мне, какие именно, просто скажите мне, СКОЛЬКО.

[Show card **T Training**, read the names and ask if the person understands: Показываете карточку **Т Подготовка**, читаете названия и спрашиваете респондента]

T1. How many of these activities have you done *in the past month*? / Сколько из этих действий вы сделали за последний месяц? [Circle answer/ Обведите ответ]

1 2 3 4 5

[Read out/ Прочтите] *Thank you, I am going to show you a card with some activities again. I am going to read their names, and then I want you to tell me how many of these activities you have done. Please, don't tell me which ones, just tell me HOW MANY.*

Спасибо, Я снова собираюсь показать вам карточку с действиями. Я собираюсь прочитать их названия, а затем я хочу, чтобы вы рассказали мне, сколько из этих действий вы выполняли. Пожалуйста, не говорите мне, какие именно, просто скажите мне, СКОЛЬКО.

[Show card **T Livelihood activities**, read the names and ask if the person understands: Показываете карточку **Т Деятельность семьи**, читаете названия и спрашиваете респондента]

T2. How many of these activities have you done *in the past year?* (September 2016-September 2017)? / Сколько из этих действий вы выполняли в прошлом году? (Сентябрь 2016- Сентябрь 2017)? [Circle answer/ Обведите ответ]

1 2 3 4 5

T3. And how many of these activities have you done *in the Autumn/Winter?* (September-February)? / Сколько из этих действий вы выполняли Осенью / Зимой? (Сентябрь-Февраль)? [Circle answer/ Обведите ответ]

1 2 3 4 5

T4. And how many of these activities have you done *in the Spring/Summer?* (March-August)? / Сколько из этих действий вы выполняли *Весной/Летом?* (Март-Август)? [Circle answer/ Обведите ответ]

1 2 3 4 5

T5. And how many of these activities have you done *for cash?* / Сколько из этих действий вы выполняли *за оплату?* [Circle answer/ Обведите ответ]

1 2 3 4 5

[Read out/ Прочтите] *Thank you, now I am going to read a list of animals, and for each animal I want you to tell me if they are a protected species or not. Please just say yes or no.*

Спасибо, теперь я собираюсь прочитать вам список животных, и я хочу, чтобы вы сказали мне, какие из этих животных онтносятся к охраняемым видам. Пожалуйста, просто отвечайте «да», «нет» или «не знаю».

T6. Which of these species is a protected species? / Какие из этих видов являются охраняемыми? [Circle answer/Обведите ответ]

Mute swan / Лебедь-шипун	Yes/Да / No/Нет / Don’t know/Не знаю
Goldeneye / Гоголь	Yes/Да / No/Нет / Don’t know/Не знаю
Lesser White-fronted goose / Пискулька	Yes/Да / No/Нет / Don’t know/Не знаю
Greylag goose / Серый гусь	Yes/Да / No/Нет / Don’t know/Не знаю
Red-breasted goose / Краснозобая казарка	Yes/Да / No/Нет / Don’t know/Не знаю

[Go to section D/Переходите к разделу D]

Section D: Hunting licences (FOR ALL RESPONDENTS)

7. a) Have you bought a licence to hunt geese? (Yes / No)
a) Вы купили лицензию на охоту на гусей? (Да / нет)

If yes/ Если да:

b) With your licence, which goose species, and how many individuals of each species can you hunt? ____________
b) С вашей лицензией, какие виды гуся и сколько людей каждого вида вы можете охотиться?
____

8. a) Does your license include other birds apart from geese? (Yes / No)
a) Включает ли ваша лицензия других птиц помимо гусей? (Да / нет)

If yes/ Если да:

b) Which species and how many individuals can you hunt for each species? ____________
b) Какие виды и сколько людей вы можете охотиться за каждым видом?
____

Section E: Opinion about cards (FOR ALL RESPONDENTS)

Раздел E: Мнение о карточках (ДЛЯ ВСЕХ РЕСПОНДЕНТОВ)

[Read out/ Прочтите] *Finally, we would like to know your opinion about the cards I showed you and the questions I asked you using these cards. For each of the topics in the table below, you should choose your answer: / Наконец, мы хотели бы узнать ваше мнение о карточках, которые я показал вам, и о вопросах, которые я задал вам, используя эти карточки. Для каждой из тем в таблице ниже вы должны выбрать свой ответ:*

	Was this easy to understand? / Были ли они легкими для понимания?	Do you feel your answer to this was anonymous? Вы чувствуете, что ваш ответ на это был анонимным?	Did you feel uncomfortable answering this? Вам было неудобно отвечать на эти вопросы?
UCT cards/ Карточки	Yes / No / Don't know Да / Нет / Не знаю	Yes / No / Don't know Да / Нет / Не знаю	Yes / No / Don't know Да / Нет / Не знаю

[Read out/ Прочтите] *Thank you for giving your time to complete this questionnaire. Your answers will help us understand how people live in rural Kazakhstan, and how can we improve our techniques when collecting information from local communities.*

Спасибо, что потратили свое время на заполнение данной анкеты. Ваши ответы помогут нам понять, как люди живут в сельской местности Казахстана и как улучшить методы сбора информации от местных сообществ.

[Write down time of completion / Запишите время окончания]

End time / Время окончания: ______________

QUESTIONS FOR ENUMERATOR / ВОПРОСЫ ДЛЯ УЧЕТЧИКА:

Was this respondent willing to answer your questions? / Был ли этот респондент готов ответить на ваши вопросы? [Circle answer/ Обведите ответ]

Very much / Да, полностью Moderately / Средне A little / Немного Not at all / Нет

How well did this person understand the questions? / Насколько хорошо этот человек понимал вопросы? [Circle answer/ Обведите ответ]

Very well / Очень хорошо Moderately / Средне A little / Немного Not at all / Нет

Do you think this person was honest when replying? / Как вы думаете, этот человек был честен, когда отвечал? [Circle answer/ Обведите ответ]

Very much / Да, полностью Moderately / Средне A little / Немного Not at all / Нет

Other comments / Другие комментарии?

____

____

____

____

____

UCT PICTURE CARDS

[Example cards modified from Whytock, Morgan, Awa, Bekokon, Abwe, Buij, Virani, Vickery, Bunnefeld, 2018. Quantifying the scale and socioeconomic drivers of bird hunting in Central African forest communities. Biol. Conserv. 218, 18–25. https://doi.org/DOI:10.1016/j.biocon.2017.11.034]

Card C: Training

**Riding in a car**	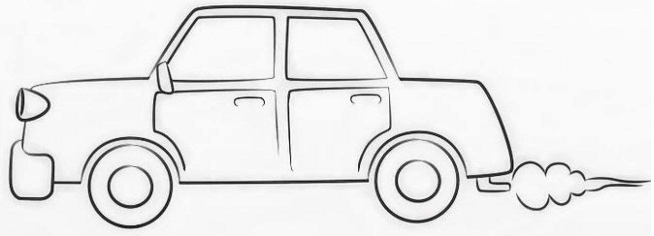
**Playing games**	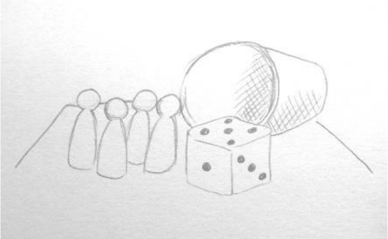
**Watching TV**	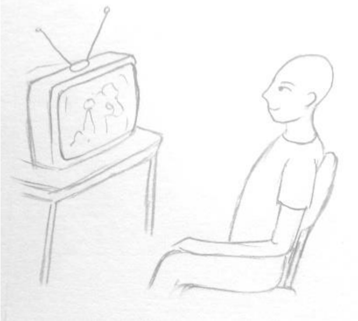
**Visiting a city**	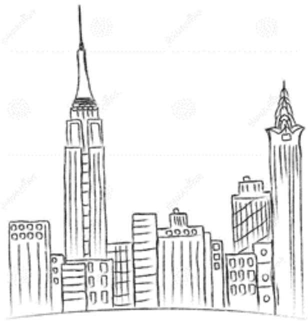

Card C: Livelihood activities

**Farming or herding**	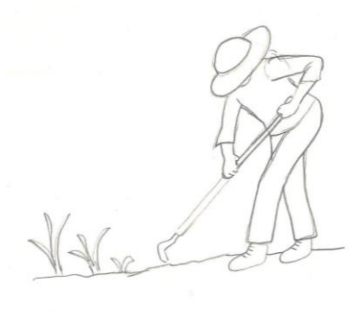
**Driving a taxi**	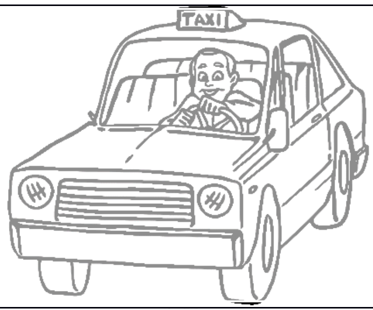
**Construction**	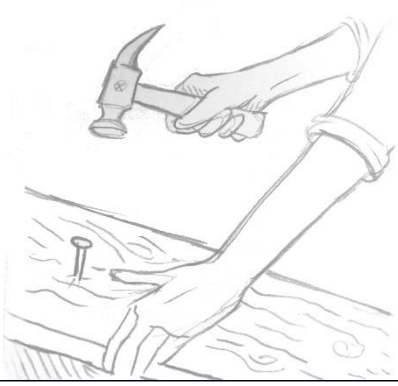
**Milking cows**	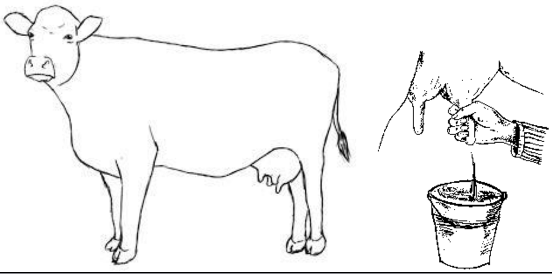

Card T: Training

**Riding in a car**	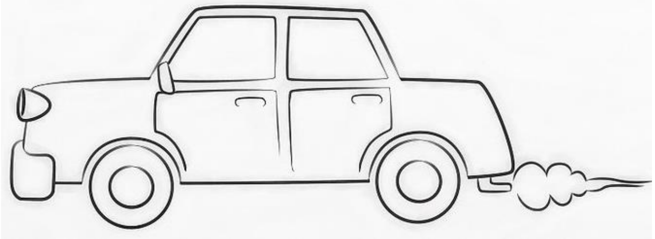
**Playing games**	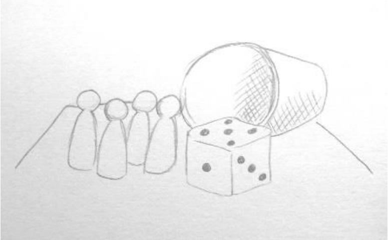
**Watching TV**	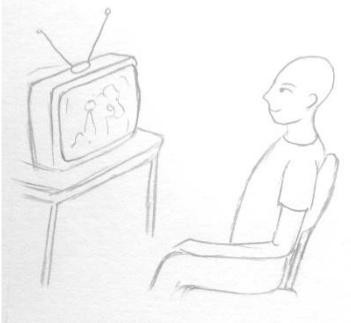
**Visiting a city**	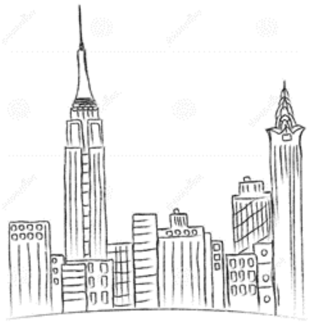
**Playing football**	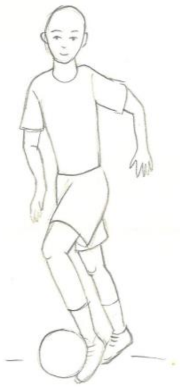

Card T: Livelihood activities

**Farming or herding**	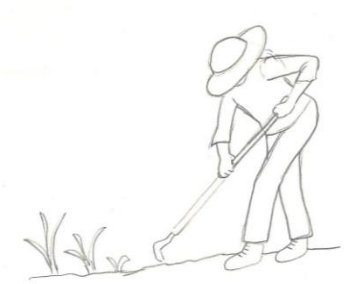
**Driving a taxi**	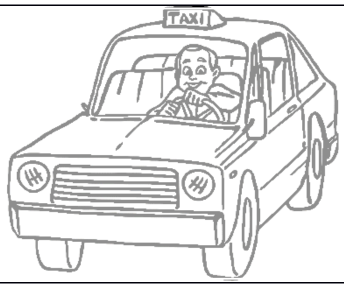
**Hunting geese**	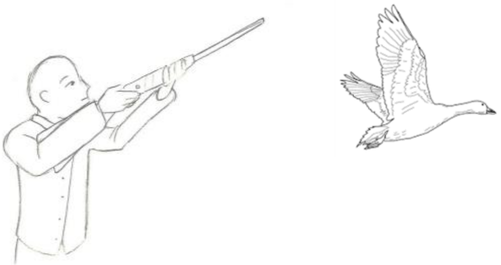
**Construction**	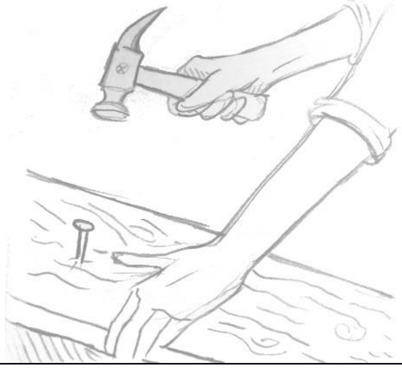
**Milking cows**	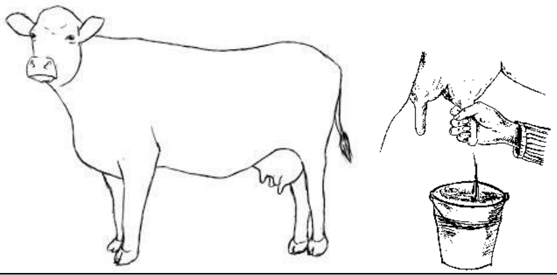
